# The macroeconomic impact of a dengue outbreak: Case studies from Thailand and Brazil

**DOI:** 10.1371/journal.pntd.0012201

**Published:** 2024-06-03

**Authors:** Kinga Marczell, Elvis García, Julie Roiz, Rameet Sachdev, Philip Towle, Jing Shen, Rosarin Sruamsiri, Bruna Mendes da Silva, Riona Hanley

**Affiliations:** 1 Evidera, Budapest, Hungary; 2 Takeda International AG, Zürich, Switzerland; 3 Evidera, London, United Kingdom; 4 Evidera, Bethesda, Maryland, United States of America; 5 Takeda Pharmaceuticals International AG, Singapore; 6 Takeda Thailand Ltd, Bangkok, Thailand; 7 Takeda Pharmaceuticals Brazil, São Paulo, Brazil; University of California Davis School of Veterinary Medicine, UNITED STATES

## Abstract

**Background:**

Dengue is spreading in (sub)tropical areas, and half of the global population is at risk. The macroeconomic impact of dengue extends beyond healthcare costs. This study evaluated the impact of dengue on gross domestic product (GDP) based on approaches tailored to two dengue-endemic countries, Thailand and Brazil, from the tourism and workforce perspectives, respectively.

**Findings:**

Because the tourism industry is a critical economic sector for Thailand, lost tourism revenues were estimated to analyze the impact of a dengue outbreak. An input-output model estimated that the direct effects (on international tourism) and indirect effects (on suppliers) of dengue on tourism reduced overall GDP by 1.43 billion US dollars (USD) (0.26%) in the outbreak year 2019. The induced effect (reduced employee income/spending) reduced Thailand’s GDP by 375 million USD (0.07%). Overall, lost tourism revenues reduced Thailand’s GDP by an estimated 1.81 billion USD (0.33%) in 2019 (3% of annual tourism revenue). An inoperability input-output model was used to analyze the effect of workforce absenteeism on GDP due to a dengue outbreak in Brazil. This model calculates the number of lost workdays associated with ambulatory and hospitalized dengue. Input was collected from state-level epidemiological and economic data for 2019. An estimated 22.4 million workdays were lost in the employed population; 39% associated with the informal sector. Lost workdays due to dengue reduced Brazil’s GDP by 876 million USD (0.05%).

**Conclusions:**

The economic costs of dengue outbreaks far surpass the direct medical costs. Dengue reduces overall GDP and inflicts national economic losses. With a high proportion of the population lacking formal employment in both countries and low income being a barrier to seeking care, dengue also poses an equity challenge. A combination of public health measures, like vector control and vaccination, against dengue is recommended to mitigate the broader economic impact of dengue.

## Author summary

Dengue is a viral illness spread by mosquitoes in Southeast Asia and South America, and about half of the world’s population is at risk. Burden-of-illness studies on the economic impact of dengue typically focus on medical costs. However, the impact of this infectious disease extends far beyond healthcare costs. This study evaluated the impact of dengue on gross domestic product (GDP) based on approaches tailored to two dengue-endemic countries, Thailand and Brazil. The analyses estimated that the dengue outbreak in 2019 had a profound impact on Thailand’s tourism industry, reducing GDP by an estimated US$1.81 billion, equivalent to 3% of annual tourism revenues or 0.33% of total GDP. Dengue also impacted Brazil’s workforce in 2019, leading to an estimated 22.4 million lost workdays, of which 39% were associated with the informal sector, and reducing GDP overall by US$876 million (0.05% of total GDP). Informal workers are vulnerable to dengue-related financial burdens, and a low income can be a barrier to seeking medical care for dengue. This study highlights the broader economic impact of dengue beyond healthcare costs and underscores the importance of a combination of public health measures, such as vector control and vaccination.

## 1. Introduction

Dengue is the most rapidly spreading mosquito-borne viral disease in the world and based on a geo-surveillance model, nearly 3.9 billion people across 124 countries are at risk of infection [1–4]. According to a 2022 report by the World Health Organization, an estimated 100–400 million dengue infections occur worldwide each year [3,5]. Of these infections, approximately 25% develop symptomatic dengue illness based on model estimation and prior studies, though this proportion can vary widely due to numerous factors [5,6]. Global warming is expected to further increase the risk of sustained dengue transmission in existing dengue-endemic countries, with spread to several non-endemic countries [7–11]. Dengue affects populations of all ages, including adults and elderly people, and has a detrimental macroeconomic impact that may encompass changes in consumption and demand across economic sectors, workforce absenteeism, healthcare costs, and government expenditures [6,12–19]. Dengue epidemics may also adversely affect tourism and foreign investment in countries that rely on tourism revenues [12]. Tropical countries have sizable informal economies involving employees with limited or no access to social security and health care, which adds to the burden posed by dengue [20–29]. Since 2021, the World Health Organization has listed the control of dengue as one of its top priorities to promote societal benefits and health equity [30].

Burden-of-illness studies tend not to fully capture the societal and economic impact of infectious diseases beyond healthcare costs [12,31]. However, the COVID-19 pandemic has highlighted the profound societal economic impact of infectious diseases. A study based on a computable general equilibrium model estimated that global GDP decreased by 1–3.4% in 2020, the deepest economic depression since the Great Depression [32]. Dengue-related illness also causes a substantial societal and economic impact in dengue-endemic countries and affects direct foreign investment. However, dengue’s macroeconomic effects are routinely overlooked in burden-of-illness studies and traditional evaluations of vaccines and other dengue-related health interventions [14,33–37]. Furthermore, because dengue is often self-managed and underreported, estimating its full disease burden has been a challenge [6,12,38]. This evidence gap results in policymakers underestimating the burden of dengue.

Understanding the macroeconomic impact of infectious diseases is important in assessing policies aimed at limiting their transmission, including vector control measures (such as chemical larvicides and fogging, releasing Wolbachia-infected mosquitoes, health education campaigns, among others) and vaccination [39–41]. Traditionally, vaccine health technology assessments have focused only on the direct healthcare costs associated with infectious diseases and vaccination [42–44]. Recently proposed frameworks, such as the Broad Assessment of Value in Vaccines (BRAVE) Initiative, are including health equity and macroeconomic gains associated with the control of infectious disease [17,42,45–48]. In line with this, macroeconomic gains were ranked fourth in terms of relevance and feasibility for inclusion in decision-making among value elements in a recent study on the value of vaccinations by experts in vaccine health technology assessments and were considered high-priority for inclusion in COVID-19 vaccine assessments by another expert panel [49,50]. However, macroeconomic gains from the value of vaccinations are still not included in the current health technology assessments in most countries [50]. A possible explanation for this might be that estimating the magnitude of the macroeconomic impact of infectious diseases and vaccination remains challenging due to methodological constraints and the need for sufficient evidence [51]. The importance of including the macroeconomic gains of vaccination for decision-making, however, depends on the prevalence and impact of the disease. For example, the COVID-19 pandemic showed the destructive effect of this infectious disease on the national economy worldwide, and a need to include macroeconomic gains from the value of vaccination in burden-of-illness studies [17,42,49,50]. Likewise, dengue-related burden-of-illness studies should also encompass the macroeconomic impact of dengue.

Dengue impacts the macroeconomy via multiple sectors, such as its detrimental effect on tourism and its capacity to disrupt the workforce due to lost workdays. Although several countries were worthy of analysis due to their significant contributions of tourism to GDP or highly endemic countries with large working-age populations, Thailand and Brazil were used as case-study examples. Southeast and South Asia had the highest global burden of dengue in the year 2019 [52]. The tourism industry is a critical economic sector in Thailand, making this country a perfect case study for demonstrating the macroeconomic impact of dengue through inter-industry linkages. Travel and tourism contributed to 20.1% of Thailand’s gross domestic product (GDP) in 2019 [53]. Notably, provinces in Thailand that were key tourist destinations also had high incidences of dengue [53–57]. Latin America has also experienced a substantial rise in dengue incidence in recent decades [58]. Brazil was selected as a case study for demonstrating the impact of dengue through lost workdays, because it reported the highest number of dengue cases worldwide in 2021–2022 [59–62], with most cases in the working-age population [63]. Furthermore, Brazil has a sizable working-age population (>69% of the total population). Dengue-related workforce absenteeism and its cascading effects along the supply chain were estimated to decrease Brazil’s GDP by 0.02% in 2013 [61]. One study based on a clinical cohort in Mexico found that approximately 55.7% of patients with dengue experienced persistent symptoms at 1 month following their acute phase of sickness, potentially further impacting productivity [64]. Notably, monthly per capita household income decreased by 28% when the primary financial contributor of the household caught dengue [27].

Many dengue cases are associated with outbreaks that are difficult to predict and can cause a disruption to the economy [13,19,65–67]. Here, we present two case studies investigating the macroeconomic impact of a dengue outbreak via two different channels, in two different dengue-endemic countries with different dominant industries contributing to GDP: Thailand and Brazil. Specifically, this study evaluated as case studies the impact of a dengue outbreak on GDP due to lost tourism revenues in Thailand and to lost workforce in Brazil, including the effect on the informal sector, which is important to capture from an equity perspective. A static input-output framework was used for both the Thailand and Brazil analysis. For Thailand, a standard input-output framework was used, capturing the shock of dengue on the demand side, while for Brazil, an inoperability input-output model capturing shock on the supply side due to the loss of workers in the workforce was used. The outbreak year 2019 was used in both studies to exclude the impact of the COVID-19 pandemic.

## 2. Methods

A static input-output framework was used to estimate the impact of a dengue outbreak on GDP in both Thailand and Brazil. The framework of input-output analysis, originally developed by Leontief [68], is designed to analyze the macroeconomic impact of a sudden change in the demand for the goods or services provided by a specific industry. In inoperability input-output models, this framework was further designed to estimate the wider economic effect of an industry directly impacted by a supply-side disruption, such as a natural disaster through the cascading effect along inter-industry linkages [69]. Input-output frameworks form the basis of many macroeconomic models employed by governments, central banks, international organizations, and academic researchers. In these organizations, the model is a widely used tool for quantifying the cascading effects of a change in the final demand of certain industries on the output and value added by economies [70–75]. These industries are linked together through their production technologies, and they use intermediate inputs from other industries during their production process to produce their own output.

**Fig 1:**
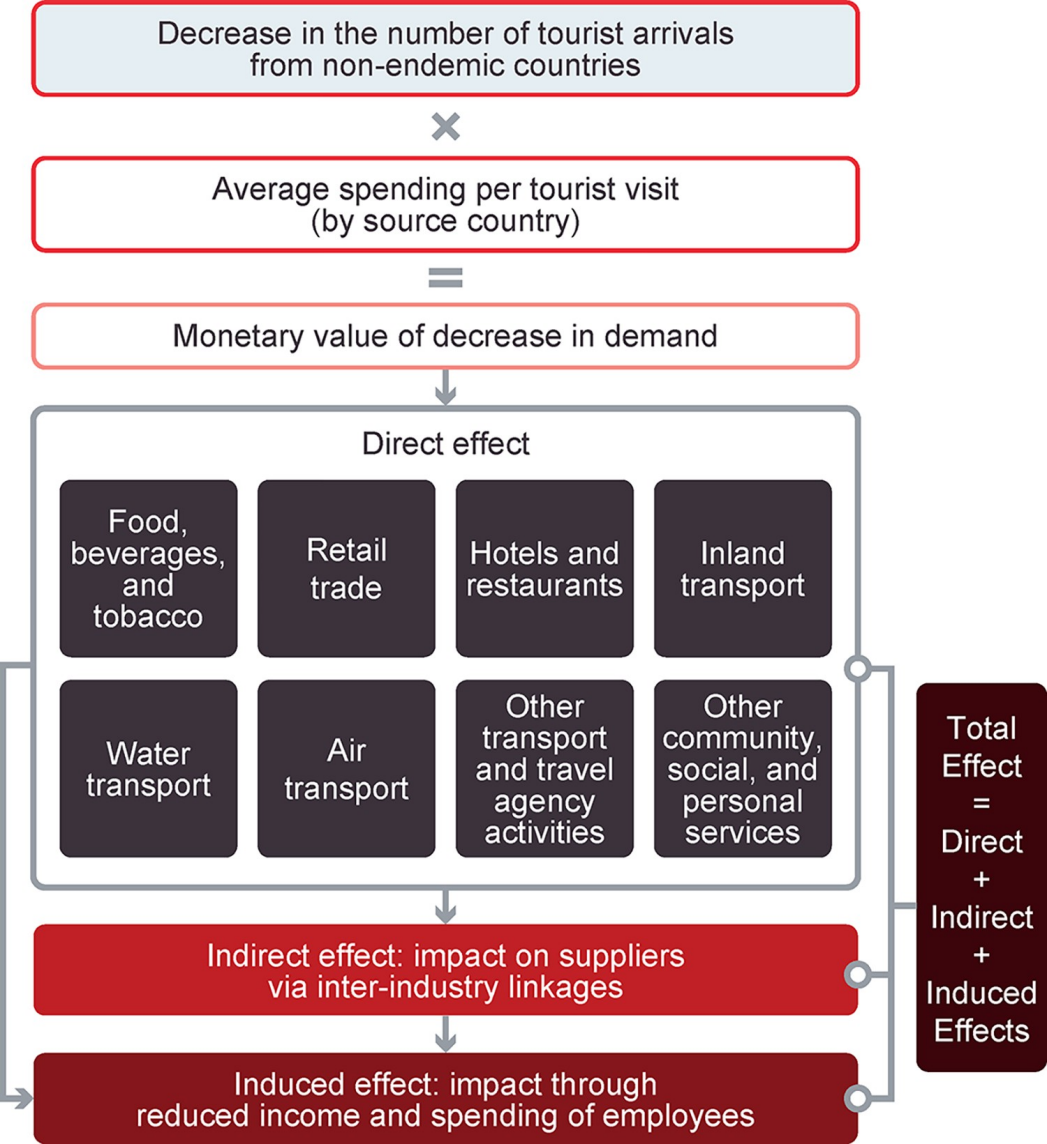
Scheme to analyze the impact of a dengue outbreak on GDP due to reduced tourism in Thailand.

In the Thailand case study, the input-output model was used to capture the sale and purchase relationships between the tourism industry and other economic sectors, and to quantify the cascading effects of changes in final demand in the tourism sector on the overall economy [68] (**Fig 1**). Calculations used as part of the input-output framework to estimate effects are provided in the **S1 Appendix**. In the model, (1) *direct effect* referred to revenue lost in the tourism sector due to a decrease in tourism following a disruption (i.e., a dengue outbreak) and its impact on Thailand’s GDP; (2) *indirect effect* was the impact that decreased demand for the product of an industry (i.e., the tourism industry) had on the demand for the products of its suppliers; and (3) *induced effect* referred to decreased household consumption due to income lost by employees working in jobs related to the tourism sector and its supply chain, further reducing the demand for goods and services.

In the Brazil case study, an inoperability input-output model (an application of the input-output framework) was used to estimate the economic effect that an industry directly impacted by disruption (e.g., a natural disaster) had on other industries due to inter-industry linkages [69]. Calculations used as part of the input-output framework are provided in the **S2 Appendix**. The inoperability input-output method, used for the Brazil analysis, has previously been applied to analyze disease outbreaks [76], including influenza [77], dengue fever [61], and, recently, COVID-19 [48].

Both case studies retrieved input-output matrices capturing the purchase and sale relationships between various industries and the household sector, industry-level GDP, and output data from the national accounts[78–80]. The analyses for both countries were carried out on the economy as a whole, which includes both the formal and informal sectors. Including the informal sector in the analyses was important because it accounts for more than 50% of total employment in Thailand and over 40% of Brazil’s workforce [20,23,29].

### 2.1 Estimating the decrease in tourism demand in Thailand

The change in tourism demand associated with a dengue outbreak was estimated based on an assumed decrease in international tourist arrivals from non-endemic countries and on statistical data on the level and structure of tourist spending patterns. Consistent with previous studies assessing the impact of a dengue outbreak on tourism, this study used estimates previously reported by Vasan et al. (2009) [36] to calculate the proportional decrease in international tourist arrivals from non-endemic countries during a dengue outbreak (**S1 Table**) [36,81,82]. Vasan et al. (2009) [36] estimated the decrease in tourist arrivals during a chikungunya outbreak on La Réunion for 2005, 2006, and 2007 to be 4%, 40%, and 17%, respectively [36]. Chikungunya has only one serotype described, and immunity against chikungunya re-infection is known to be long-lasting and maybe even life-long. Chikungunya epidemics are, therefore, rare but intense in non-immune populations [83]. Epidemiological and tourism data show substantial overlap between popular tourist destinations and dengue cases in Thailand [56,57]. Popular tourist destinations and their surrounding provinces in the North (Chiang Mai, Chiang Rai), South (Songkhla, Phang Nga), and Central (Bangkok, Chonburi) regions of Thailand reflect endemic dengue; the cases are present across years, and especially hard-hit during outbreak years [56]. However, La Réunion is a small island; thus, an even larger fraction of the country’s tourist destinations might be impacted by an infectious disease outbreak than it would in Thailand. To account for the difference between chikungunya and dengue, and the geographical difference between the two countries, we used the lowest of the three values (4%) measured by Vasan et al. (2009) [36]. To reflect the large uncertainty in this important input parameter, we conducted scenario analyses assuming alternative values for the percentage decrease in the number of tourist arrivals from non-endemic countries. The number of tourist arrivals from dengue endemic countries was assumed to be unaffected by the outbreak. The analysis assumed no impact on domestic tourism because of a lack of published supporting evidence. The total revenue generated from international tourists in Thailand was 61,572 million USD in 2019, of which 46,352 million USD was from tourists from non-endemic countries [54].

Data regarding the level and structure of tourist spending were from the Thailand Ministry of Tourism and Sports exit surveys [84]; more information about these data is provided in **S1 Appendix**. These self-reported data included expenditures in the formal and informal economy, but the proportion of formal versus informal spending was unknown. The spending categories were mapped to various industries (**S1 Table** and **S1 Appendix)**, and scenarios were conducted to explore differences in the spending distribution across industries (**S2 Table**). The impact of Thailand’s dengue-endemic status on tourism demand from all countries (endemic and non-endemic countries) was evaluated in a separate, exploratory analysis (**S1 and S3 Tables and S1 Appendix**).

### 2.2 Estimating the number of lost workdays and inoperability in Brazil

**Fig 2:**
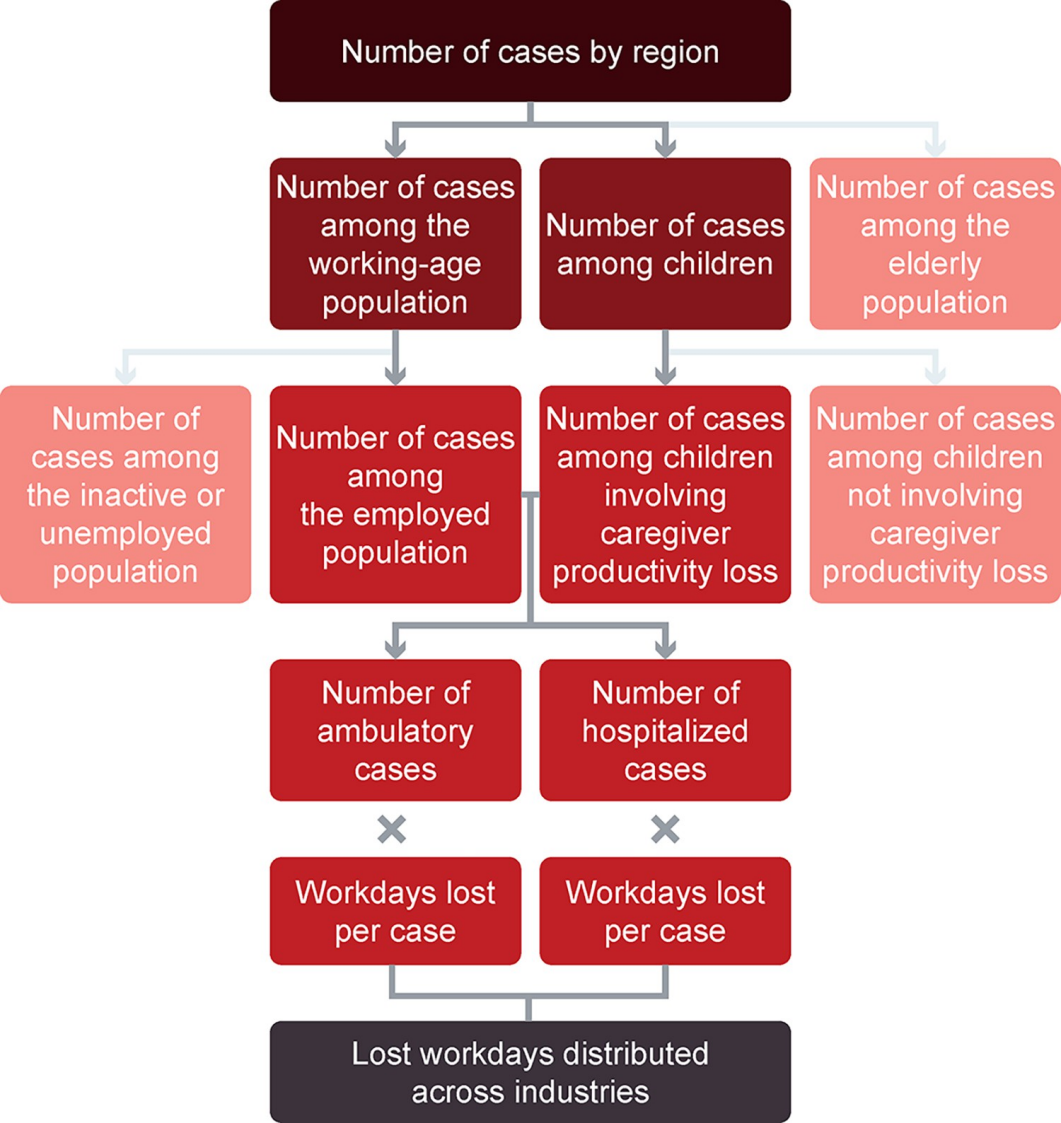
Flow chart to estimate lost workdays in 2019 due to a dengue outbreak in Brazil.

Sector inoperability is expressed as the percentage of output lost in each economic sector due to the reduced availability of the workforce because of dengue. The direct change in workforce capacity was calculated as the percentage of workdays lost in different economic sectors on the basis of the method proposed by Santos et al. (2013) [77]. The analysis estimated the number of lost workdays in 2019 due to a dengue outbreak in Brazil, and the associated industry inoperability, in three stages. Firstly, the number of ambulatory and hospitalized dengue cases among employees and children were calculated. Secondly, dengue case numbers were translated to lost workdays in the employed population. Finally, the lost workdays were associated with specific industries to determine the share of lost workdays and output for each industry (**Fig 2**).

State-level data on dengue case numbers from the Notifiable Diseases Information System from Brazil’s Ministry of Health, and information from the Ministry of Health on the age distribution of dengue cases, were used to calculate the number of dengue cases in children (ages 0–14 years) and the working-age population (ages 15–64 years) in each region of Brazil (**S4 Table**). Furthermore, dengue cases included in this analysis received healthcare treatment. Incidence data were collected from Sistema de Informação de Agravo de Notificação (SINAN) [63], and the percentage of hospitalized cases is from SIH/SUS. Both sources include cases reported by healthcare institutions and include no self-reported cases [85,86]. The 2019 state-level population estimates published by the Brazilian Institute of Geography and Statistics and information on the number of employees from the National Household Sample Survey were used to determine the number of dengue cases among employees [78]. The number of dengue cases was categorized by ambulatory and hospitalized healthcare settings based on age-specific hospitalization rates calculated from the Ministry of Health data.

To account for dengue underreporting, an expansion factor (the number by which reported cases should be multiplied to estimate the actual number of cases) was applied to provide a more accurate estimate. Expansion factors of 2.03 and 4.06 were applied for the hospitalized and ambulatory settings, respectively [87,88]. More information on the expansion factors used is presented in **S2 Appendix**. Lost workdays included days when employees were absent due to sickness or caregiving responsibilities for children. Data regarding the number of children with both parents in employment were not available. Therefore, to determine the proportion of pediatric cases associated with a caregiver having to forgo work, this study used the proportion of working-age men and women in formal or informal employment and chose the lower value of the two, which corresponded to women (47%) [78,89]. Note that this proportion might be an overestimate because, in some cases, friends/family members or part-time workers cover caregiving responsibilities for pediatric cases. No lost workdays were assumed associated with dengue cases occurring in the unemployed and inactive adult population. The estimated workdays lost per dengue case for employees were 10.7 days for hospitalized cases and 7.1 days for ambulatory cases, as reported by Suaya et al. (2009) [38].

For each state in Brazil, the sum of lost workdays for patients and caregivers was distributed across industries in proportion to the distribution of employees across industries according to the Central Register of Companies (CEMPRE) datasets. Further details on this calculation are described in **S3 Appendix**. The GDP impact was derived from the estimated number of lost workdays using the inoperability input-output model, as described in **S2 Appendix**.

## 3. Results

### 3.1 Thailand–Effect of a dengue outbreak on GDP due to reduced tourist arrivals

A 4% decrease in tourist arrivals from non-endemic countries in 2019 due to an outbreak of dengue reduced Thailand’s direct gross tourism revenues by 1.85 billion USD, which was equivalent to 3% of the annual total tourism revenue. The estimated distribution of tourism revenue across industries is presented in **S5 Table**. The direct GDP loss was estimated to be 716 million USD (0.13% of total GDP) in the dengue outbreak year 2019. Cascading effects along the supply chain resulted in a 718 million USD (0.13% of total GDP) decrease in GDP created by industries supporting the tourism sector. The estimated distribution of tourism revenue across industries is presented in **S5 Table**. The combined direct and indirect effects were associated with an approximately 1.43 billion USD (0.26% of total GDP) decrease in GDP. The reduced demand for labor decreased the total value of employee compensation by 453 million USD (0.27% of total GDP, direct and indirect effects combined), leading to decreased household consumption. The decrease in household consumption further contributed to the cascading impact of a dengue outbreak on all industries. This induced effect reduced GDP by 375 million USD (0.07% of total GDP) in 2019. Overall, Thailand’s GDP decreased by an estimated 1.81 billion USD based on the modeled 4% decrease in tourism, equivalent to 0.33% of Thailand’s GDP in 2019 (**Table 1**). As part of Thailand’s lost GDP, the compensation of employees decreased by 0.34%, causing households to lose 566 million USD (**Table 1**). The 1,854 million USD decrease in exports corresponding to tourism revenues was partly counterbalanced in the trade balance by an increase in imports. The 176 million USD in imports directly used by the tourism sector to produce the goods and services for which the demand has disappeared due to a dengue outbreak were assumed to be canceled in the input-output model framework. Another 244 million USD of imports on behalf of the tourism sector’s suppliers would also become superfluous. In response to a wage decrease, household demand for imported goods would also be lower, by an estimated 101 million USD. Overall, a dengue outbreak is expected to deteriorate the trade balance by 1,333 million USD or by 1.55% (**Table 1**).

**Table 1:** Estimated effect of a dengue outbreak in Thailand through reduced tourist arrivals from non-endemic countries on macroeconomic outcomes in 2019.

	Effect (million USD)					Effect (%)				
	Direct effect	Indirect effect	Induced effect	Direct + indirect effect	Total	Direct	Indirect	Induced	Direct + indirect	Total
GDP	−716	−718	−375	−1,433	−1,808	−0.13	−0.13	−0.07	−0.26	−0.33
Compensation of employees	−242	−211	−113	−453	−566	−0.14	−0.13	−0.07	−0.27	−0.34
Trade balance	−1,678	244	101	−1,433	−1,333	−1.95	0.28	0.12	−1.67	−1.55
Import	−176	−244	−101	−421	−521	−0.09	−0.12	−0.05	−0.20	−0.25
Export	−1,854	0	0	−1,854	−1,854	−0.63	0.00	0.00	−0.63	−0.63

GDP, gross domestic product; USD, United States dollar.

Alternative estimates for the estimated change in GDP based on alternative input values for the decrease in tourist arrivals from non-endemic countries are also shown in Table 2.

**Table 2:** Estimated change in Thailand’s GDP associated with a dengue outbreak due to reduced tourist arrivals from non-endemic countries in 2019 using alternative input values for reduced tourist arrivals.

		Estimated change in GDP				
Proportional reduction in tourist arrivals from non-endemic countries		Direct effect	Indirect effect	Induced effect	Direct + indirect effect	Total effect
0.8%	Million USD	−143	−144	−75	−287	−362
	Percentage	−0.03%	−0.03%	−0.01%	−0.05%	−0.07%
1%	Million USD	−179	−179	−94	−358	−452
	Percentage	−0.03%	−0.03%	−0.02%	−0.07%	−0.08%
2%	Million USD	−358	−359	−187	−717	−904
	Percentage	−0.07%	−0.07%	−0.03%	−0.13%	−0.17%
**Base-case: 4%**	**Million USD**	−**716**	−**718**	−**375**	−**1,433**	−**1,808**
	**Percentage**	−**0.13%**	−**0.13%**	−**0.07%**	−**0.26%**	−**0.33%**
5%	Million USD	−895	−897	−469	−1,792	−2,261
	Percentage	−0.16%	−0.16%	−0.09%	−0.33%	−0.42%
10%	Million USD	−1,789	−1,795	−937	−3,584	−4,521
	Percentage	−0.33%	−0.33%	−0.17%	−0.66%	−0.83%

GDP, gross domestic product; USD, United States dollar.

**Fig 3:**
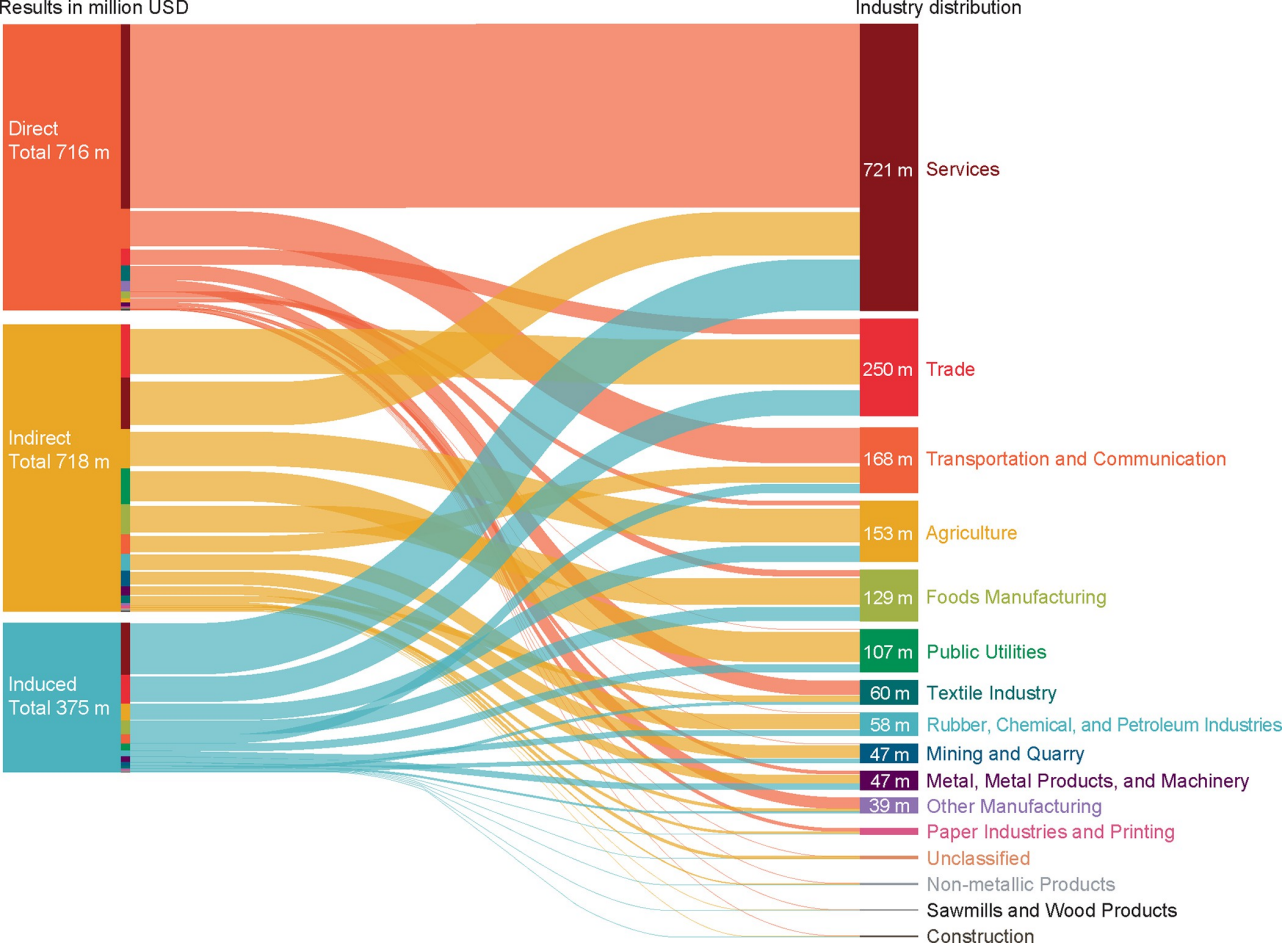
Decrease in Thailand’s GDP due to reduced tourist arrivals during a dengue outbreak. *Direct effect* refers to lost income in the tourism sector. *Indirect effect* is the impact that a decrease in demand for the product of one industry has on the demand for the products of its suppliers. *Induced effect* is the impact on household consumption due to income lost by employees working in jobs related to the tourism sector and its supply chain, further decreasing the demand for goods and services. All estimates are in United States dollars (USD). GDP, gross domestic product.

Industries are impacted via direct, indirect, and induced effects of a dengue outbreak. As observed in **Fig 3**, which provides an aggregated and industry-specific visual representation of the cascading implications of a dengue shock on the economy, the services sector was by far the most impacted (direct, indirect, and induced) because it contributes most substantially to tourism revenues. Other industries directly impacted by the decrease in tourism included transportation and communication, trade, and textile industry (**Fig 3** and **Table 3**).

**Table 3:** Distribution of estimated direct, indirect, induced, and total effects on Thailand’s GDP by industry associated with a dengue outbreak due to reduced tourist arrivals from non-endemic countries in 2019.

Industry	Effect on GDP, %			
	Direct effect	Indirect effect^a^	Induced effect^a^	Total effect^a^
Services	65	18	34	40
Trade	6	18	20	14
Transportation and Communication	14	7	6	9
Agriculture	2	14	11	8
Foods Manufacturing	2	11	10	7
Public Utilities	0	12	5	6
Textile Industry	5	2	1	3
Rubber, Chemical, and Petroleum Industries	0	6	4	3
Mining and Quarry	0	5	3	3
Metal, Metal products, and Machinery	1	3	4	3
Other Manufacturing	4	1	1	2
Paper Industries and Printing	1	1	0	1
Unclassified	0	0	0	0
Non-metallic Products	0	0	0	0
Sawmills and Wood Products	0	0	0	0
Construction	0	0	0	0
Total^a^	100	100	100	100

GDP, gross domestic product.

^a^The table presents the share of each industry in the estimated lost GDP rounded to zero digits. The effect of all industries combined is 100% per definition.

A sensitivity analysis was performed to investigate whether the results were sensitive to the distribution of spending and how spending was divided across industries (**S2 Table**). The estimated GDP decrease was 0.32% - 0.34% for all scenarios investigated (base case was 0.33%).

### 3.2 Brazil–Effect of a dengue outbreak on GDP through disrupting production

This study estimated that 2.7 million ambulatory dengue cases occurred in the employed population aged 15–64 years in 2019 (**S2 Table**). The number of hospitalized cases was approximately 70,000 in the working-age population (aged 15–64 years), including 41,000 cases among the employed population. Approximately 43,000 cases were reported in the remaining population, including children and those aged 65 years or more (**S2 Table**).

The total number of lost workdays was nearly 22.4 million; a detailed industry distribution breakdown is provided in the **S1 Fig**. Of these 22.4 million lost workdays, approximately 19.9 million were lost due to ambulatory and hospitalized dengue cases in the employed population, and 2.5 million due to caregiving responsibilities for sick children. Combining the geographical distribution data for the total number of lost workdays and regional data for the ratio of employees in the formal versus informal sectors, the model estimated that 39% of lost workdays were associated with employees in the informal sector [89].

**Fig 4:**
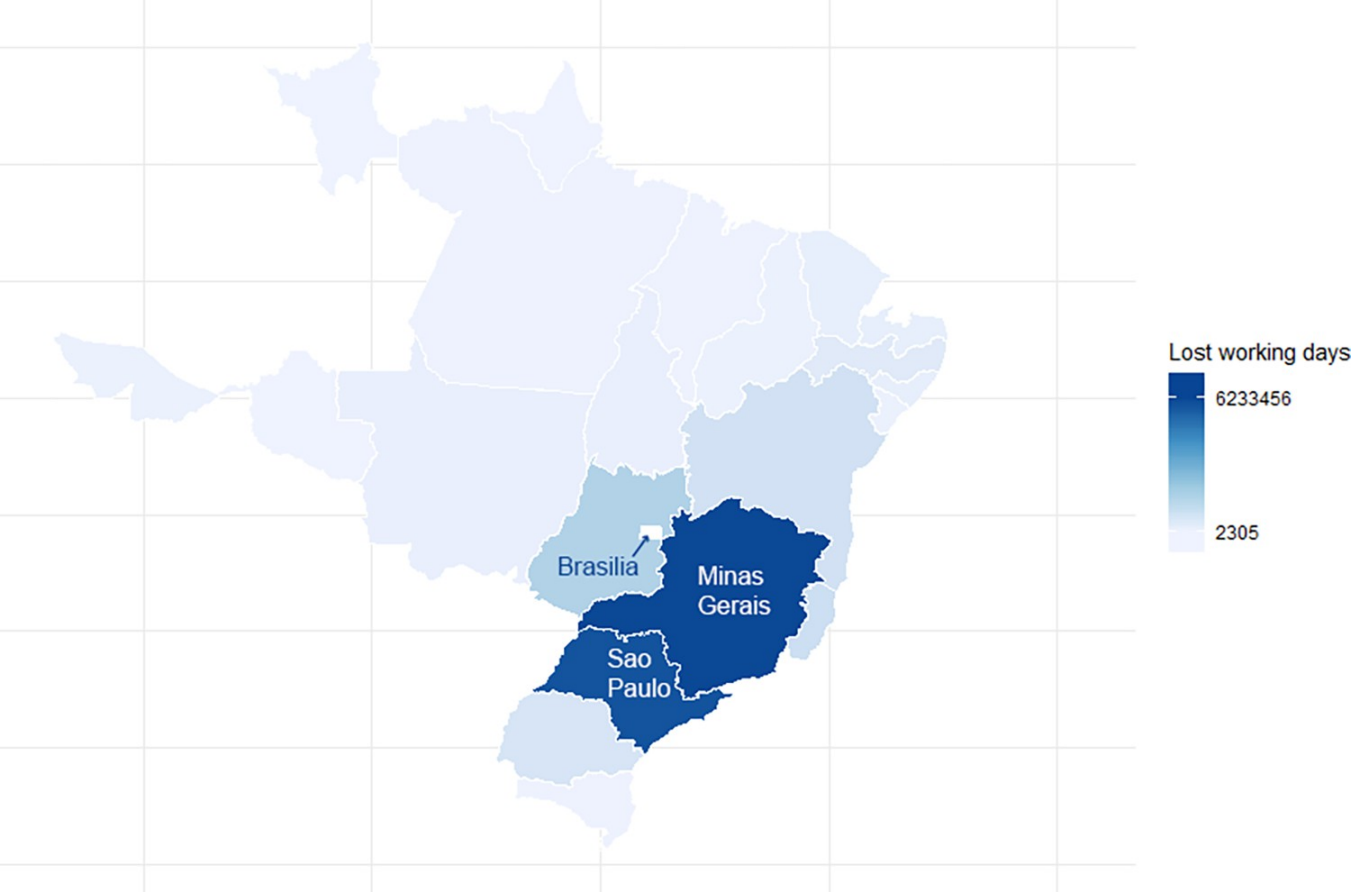
Estimated number of lost workdays due to a dengue outbreak by region in Brazil in 2019. The number of lost workdays was estimated based on the ambulatory and hospitalized numbers of dengue cases among the formally and informally employed population and children as described in the Methods section. The map was created using geobr [90] based on shapefiles provided by the Brazilian Institute of Geography and Statistics (IBGE) [91].

The geographical distribution of estimated lost workdays due to a dengue outbreak was imbalanced (**Fig 4**); Minas Gerais and São Paulo accounted for approximately 60% of all dengue cases, potentially due to the high dengue incidence in Minas Gerais and the large employed population in São Paulo.

The estimated effect of a dengue outbreak on inoperability was highest in industries with a large share of dengue-impacted workforce and industries (e.g., education, which accounted for almost 0.09% of lost output) (**S2 Fig**). Inoperability in highly impacted industries had cascading effects on the rest of the economy through supply chain linkages. Accounting for the indirect effect on inoperability led to a more equal distribution of impact, with inoperability levels of 0.06–0.09% for the five most impacted industries. A breakdown of inoperability by industry is provided in **S2 Fig**.

The estimated impact of a dengue outbreak on GDP was −876 million USD, equivalent to 0.05% of Brazil’s GDP. Industries that contribute substantially to baseline GDP had the most impact on GDP due to dengue (**S3 Fig**).

Scenario analyses for the Brazil study were conducted to understand the impact of parameter uncertainties on the results; the results are presented in Table 4. Assuming the number of lost working days per ambulatory and hospitalized cases to equal the mean minus/plus two standard errors of the value reported by Suaya et al. (2009) [38] led to an estimated GDP decrease of 0.04% and 0.05%, respectively (**Table 4**). The results were not overly sensitive to a 20% change in hospitalization rate or to a 20% change in the percentage of children requiring a caregiver to forgo work either (**Table 4**). Using alternative expansion factors based on Martelli et al. (2015); 1.6 days for hospitalized cases and 3.2 for ambulatory cases (2.03 and 4.06 in the base case, respectively) [88]) estimated a total GDP decrease of 0.04%, compared to 0.05% in base case.

**Table 4:** The impact of alternative input values for input parameters on GDP.

	Effect (million USD)			Effect (%)		
	Direct	Indirect	Total	Direct	Indirect	Total
Base case	−568	−308	−876	−0.03	−0.02	−0.05
Lower lost days (-2 SE from Suaya et al. [38], i.e. 6.4 days ambulatory, 9.5 days hospitalized)	−512	−278	−790	−0.03	−0.01	−0.04
Higher lost days (+2 SE from Suaya et al. [38], i.e. 7.8 days ambulatory, 11.9 days hospitalized)	−624	−338	−963	-0.03	-0.02	-0.05
Lower hospitalization rate within reported cases (-20%, i.e. 4.1% for adults and 2.3% for children)	−569	−308	−878	−0.03	−0.02	−0.05
Higher hospitalization rate within reported cases (+20%, i.e. 6.2% for adults and 3.5% for children)	−567	−307	−875	−0.03	−0.02	−0.05
Less children requiring caregivers (-20%, i.e. 37.6%)	−555	−301	−856	−0.03	−0.02	−0.05
Expansion factors from Martelli et al. [88] (1.6 days for hospitalized, 3.2 days for ambulatory)	−448	−243	−691	−0.02	−0.01	−0.04

SE, standard error; USD, United States dollar.

## 4. Discussion

While the detrimental impact of dengue fever on macroeconomic outcomes is frequently mentioned in the literature [6,13–18,67], published quantitative estimates of this effect are scarce. This analysis contributes to the literature by assessing the effect of reduced tourism revenue on Thailand’s GDP and increased workforce absenteeism on Brazil’s GDP due to a dengue outbreak in 2019 (prior to the COVID-19 pandemic). Our analyses showed that dengue has a profound effect on the tourism sector in Thailand and the workforce in Brazil.

As acknowledged in the literature, accurate estimation of dengue’s impact on tourism is challenging, due to the lack of data availability and the difficulty of defining the time period and exact geographical area impacted by an outbreak [6]. Evidence supporting dengue as a reason for deterring tourism is typically anecdotal, citing company annual reports from airlines, travel agencies, tourism consultancies, and communications from public authorities [81,82]. Only a few scientific studies have systemically quantified the relationship between tourism and dengue incidence [36,92,93]. Consequently, the current analysis relied on the scarce data available to assist in generating exploratory evidence of dengue’s impact on tourism and contribute towards this evidence gap. Furthermore, the relationship between dengue incidence and tourism revenue is under researched relative to its importance in the wider economic impact. Nevertheless, the estimates from this study need to be interpreted with caution as input parameters are subject to high degrees of uncertainty, as reported by Vasan et al. (2009) [36].

This study analyzed dengue’s impact on Thailand’s GDP based on estimates for a decrease in tourism arrivals in Thailand from non-endemic countries reported by Vasan et al. (2009) [36]. A study based on 2008 prices estimated that a decline in tourist arrivals from non-endemic countries translated to a 363 million USD loss in tourism revenue for Thailand, equivalent to 0.12% of GDP [82]. Both the study based on 2008 prices and the present study used the lowest estimated decrease in international tourist arrivals from non-endemic countries reported by Vasan et al. (2009) [36]. However, Thailand’s tourism revenue almost quadrupled from 2006 to 2019 [84]. Based on 2019 data, our study predicted lost tourism revenue of over 1.8 billion USD (0.33% of total GDP) due to a dengue outbreak.

In Thailand, the estimated macroeconomic impact of dengue is substantial compared with the direct costs of dengue recognized in burden-of-illness studies. Shepard et al. (2016) [15] reported the overall annual aggregated cost due to dengue incidence in Thailand was 424.8 million USD in 2013 prices, including the direct medical costs of cases admitted to hospital and ambulatory care (349.4 million USD), direct non-medical costs corresponding to dengue episodes treated outside the professional healthcare sector (3.8 million USD), and indirect costs associated with time lost because of illness or care (71.6 million USD) [15]. The total healthcare and medical cost reported by Shepard et al. (2016) [15] in 2019 prices was 435.9 million USD. This cost burden, representative of an average year, was adjusted by the ratio of the 2019 incidence (197.27 per 100,000) and the average incidence between 2011–2019 (141.34 per 100,000), as extracted from Thailand’s Ministry of Public Health data to estimate the cost burden in the outbreak year 2019 [94,95]. Applying this adjustment factor of 1.4 resulted in an adjusted cost burden of 608.4 million USD. However, the present study estimated the total effect of a dengue outbreak on GDP to be more than 1.8 billion USD, which included costs beyond the healthcare burden of dengue, suggesting that the financial costs associated with tourism losses during an outbreak year surpasses financial costs associated with illness.

In Brazil, the estimated macroeconomic impact of dengue is also sizable compared with the costs of dengue estimated in burden-of-illness studies. In a previously reported prospective, multicenter, observational study in Brazil the average aggregate direct medical costs were 164 million USD (243 million USD in 2019 prices) in 2012–2013, a time period with an average incidence close to the incidence observed in 2019 [63,88]. The same estimate increased to 447 million USD (663 million USD in 2019 prices) after adjustment for underreporting. Other studies estimated the total societal cost (including the economic value of human life associated with death cases) of dengue in Brazil to be 878 million USD (1.7 billion USD in 2019 prices) [18] and 728 million USD (1.1 billion USD in 2019 prices) [15]. Our estimated macroeconomic impact of dengue, not including medical costs and the economic value of lost lives, is 876 million USD.

Estimates for workforce losses due to dengue align with Montibeler et al. (2018) [61], who estimated that a dengue-related workforce loss and its cascading effects caused a 0.02% decrease in Brazil’s GDP, an equivalent of 361 million USD in 2019 prices. Nonetheless, the present study used more recent data accounting for geographical variation in dengue incidence, employment level, industry structure, and underreporting [61]. Furthermore, the study from Montibeler et al. (2018) [61] was based on the number of reported dengue cases and did not account for dengue underreporting. Excluding the impact of underreporting, the model in the present study provides comparable results: a total GDP impact of 221 million USD (0.01%). However, the inclusion of underreporting results in a substantial increase to 876 million USD (0.05%). Analyses with the inclusion of underreporting are important; accurate estimation of dengue poses a substantial challenge because a high proportion of those infected self-manage their symptoms and thus may not be recorded by epidemiological surveillance [6]. Moreover, a large proportion of Brazil’s workforce consists of the informal economy, meaning workers may be hesitant to engage with healthcare services. These factors combined eventually limit the reporting capabilities by healthcare services of dengue infections [22].

The inclusion of both formal and informal workers in the analyses is important, given the substantial contribution of the informal economies toward Thailand and Brazil’s GDP, and underscores the importance of health equity. In Thailand, the GDP created by the informal tourism economy, the employees of which have worse access to paid sick leave [96,97], is estimated to be nearly half of that created in the formal tourism economy [20,21]. In Brazil, the present study estimated that 39% of lost workdays were associated with employees in the informal sector, a similar magnitude as the share of employees in the informal sector (41%) [22], suggesting that those working in the informal economy are similarly impacted as formal workers and bear an important share of the overall burden.

Urban areas are reported to have higher dengue seroprevalence and incidence than rural areas in India [98–101], Northeastern Thailand, Southern Laos [102], and Mexico [103]. Furthermore, higher-income areas associated with urbanization are linked to increased dengue transmission in Brazil [104]. Nonetheless, rural areas not previously impacted are also experiencing dengue outbreaks, likely due to an increased infrastructure between rural and urban cities [105]. Although the literature suggests variation in the urban and rural impact of dengue by country and geographic profile, a multi-country systematic literature review covering 106 studies and 347 estimates suggests that dengue is no longer predominantly an urban disease; the incidence in rural areas has grown over time, and some studies show similar impact of dengue between urban and rural settings [13,106–108].

A systematic literature review exploring 12 studies across Southeast Asia and Latin America found mixed results between studies on associations between different poverty indicators and dengue burden [109]. Other studies suggest poverty may lead to increased dengue severity, though this may also be due to other covariates specific to the geographic region [110–112]. A recent systematic literature review found that lower income and rural settings were among several barriers to seeking healthcare for dengue [113]. Delay in care-seeking can lead to worse health outcomes, including severe symptoms and dengue-related death [113].

The COVID-19 pandemic has also highlighted the economic and societal benefits of preventing infectious disease outbreaks [17,47,48,114]. As noted in the introduction, there is a general shift toward focusing more closely on global health equity, highlighted by recent value frameworks such as BRAVE, which encourages consideration of health equity as part of health technology assessments, and also by the WHO prioritizing health equity as part of their mission [115,116].

There are limitations in the input-output model as used in the analyses, in that prices are fixed and only the quantities of products create change because of demand shocks, implying an infinite elasticity of supply. Furthermore, there is no substitution between goods and services due to the assumption of fixed technology [70,117], and products and services that can replace each other could affect the magnitude of the effect estimate. For the Thailand study, the induced effects were calculated based on the assumption that the savings rate of households is unaffected by the change in tourism demand. Families of patients previously hospitalized with dengue in Thailand lost more than their average monthly income (61 USD), which supports the assumption that households are forced to decrease their consumption in response to a substantial decrease in their wage income [25,118,119]. However, these assumptions are more applicable to cases of a temporary shock (e.g., a dengue outbreak) rather than to any long-term changes due to the impact of endemic dengue, when substitution between goods and services would mitigate the impact [70]. Furthermore, the analysis did not include Thailand’s competitors in the international tourism market. The dengue-endemic status of these countries may impact the competitive advantage of Thailand, especially if they experience changes in dengue incidence similar to those in Thailand.

The inoperability input-output model used for the Brazil study shares the limitations inherent to input-output frameworks. Inoperability input-output models are used to quantify the effect of disruptions in interdependent sectors during disaster impact assessment, though some methodological aspects of these models are subject to criticism, including the demand-driven propagation path for supply-side shocks [120]. The potential of the initial shock to create spill-over effects in the economy can be mitigated by the ability of firms to adjust their production process as a response, e.g. by replacement of lost workforce with reallocations of other resources or more reliance on available workers. This was not formally considered in the analysis due to lack of data on the flexibility of firms to adjust to these shocks. Computable general equilibrium (CGE) models have relaxed many of these limitations by allowing production technologies and prices of products, services, and labor to adjust in response to the shock analyzed. CGE models have also previously been used for analyzing the impact of infectious diseases [121–125], and CGE models were considered for the current analysis. However, CGE models have their own limitations, including being complex, computationally intensive, and requiring substantially more data. Therefore, the input-output framework was selected for these case studies, and future analyses are recommended to build upon the current scope and use this work as a basis of a CGE model.

The results are also subject to uncertainty due to ambiguity in the input data. The decline in tourist arrivals due to dengue in Thailand was difficult to determine precisely due to a lack of published evidence [6]. Furthermore, dengue incidence in Brazil varies greatly by state and year; the total macroeconomic impact may vary over time. The analysis reflects the dengue incidence in 2019 and aligns with the economic data used in the present study. Predicted expansion factors also vary due to the quality of reported case data, healthcare systems, available surveillance data, and reporting mechanisms, as well as annual variation of incidence [126,127]. Furthermore, if surveillance systems were to improve, the estimated expansion factors used in this study may be subject to overestimation. The percentage of pediatric dengue cases requiring a caregiver to forgo work is highly uncertain due to the lack of data. The resulting estimated share of lost workdays borne by the patient within all lost workdays (89% for ambulatory and 84% for hospitalized cases) is somewhat higher that what was reported in the literature (77% for ambulatory and 76% for hospitalized cases) [38], suggesting a possible underestimation of the burden. This may be partially or entirely due to not accounting for caregiver burden associated with adult dengue cases.

In summary, the data presented here contribute to the limited evidence available on the macroeconomic impact of dengue beyond healthcare costs in Thailand and Brazil. The estimates show that the economic consequences of dengue far surpass the direct medical costs associated with the disease.

Other countries with a high dengue incidence and a high contribution of tourism to GDP (such as Mexico, Cambodia, and the Philippines) may experience a similarly substantial macroeconomic burden due to dengue (**S4 Fig**), as also emphasized by Mavalankar et al. (2009) [82]. Additionally, the impact of a dengue outbreak causing temporary disruption in productivity is relevant for all dengue-endemic countries. Although the current analysis can be applicable to the countries mentioned above at a conceptual level, due to variations in local economic structures, disease patterns, and healthcare resources, the magnitude of the estimates is expected to vary across countries. This study adds to the literature by emphasizing a broader value assessment of vaccination, consistent with the BRAVE guidelines, which include macroeconomic gains among the elements of vaccination’s societal impact [42]. The results of this study also highlight that policies aimed at mitigating the burden of infectious diseases, such as vector control and vaccination, need to be designed based on a thorough understanding of the impact of these diseases on the economy and society.

## Supporting information

S1 Appendix Input-output model framework for Thailand. Mapping of spending categories. Statistical Information on Tourist Expenditure and the Informal Economy. Effect of dengue-endemic status on GDP due to reduced tourist arrivals.
(DOCX)

S2 Appendix Inoperability and impact on Brazil’s GDP. Estimation of lost workdays in the informal sector.
(DOCX)

S3 Appendix Analysis of the CEMPRE datasets. (DOCX)

S1 Fig Estimated number of lost workdays due to a dengue outbreak by industry in Brazil in 2019. (TIF)

S2 Fig Estimated direct and indirect effect of a dengue outbreak on inoperability due to productivity loss in Brazil in 2019. (TIF)

S3 Fig Estimated direct and indirect impact of a dengue outbreak on GDP due to productivity loss in Brazil in 2019. (TIF)

S4 Fig Dengue-endemic countries with high travel and tourism contributions to GDP. (TIF)

S1 Table Input data for Thailand. (DOCX)

S2 Table Sensitivity analyses exploring alternative distributions of tourist spendings across industries in Thailand. (DOCX)

S3 Table Estimated effect of reduced tourist arrivals due to endemic dengue on macroeconomic outcomes in 2019. (DOCX)

S4 Table Input data for Brazil. (DOCX)

S5 Table Estimated industry distribution of international tourism revenue of Thailand in 2019. (DOCX)
