# Supplementary material for: The macroeconomic impact of a dengue outbreak: Case studies from Thailand and Brazil
Source: PLoS Negl Trop Dis. 2024 Jun 3;18(6):e0012201. doi: 10.1371/journal.pntd.0012201 (PMC11175482; doi:10.1371/journal.pntd.0012201)
Supplement: S1 Appendix — Mapping of spending categories. Statistical Information on Tourist Expenditure and the Informal Economy. Effect of dengue-endemic status on GDP due to reduced tourist arrivals. (DOCX) [file pntd.0012201.s001.docx]

## S1 Appendix

### Input–output model framework for Thailand

Output created by one industry will partly serve as an intermediate input for other industries and will partly be used to satisfy the final demand. Final demand encompasses household consumption, investment, government expenditure, and net export. This relationship can be expressed as:

*Equation 1*

***x*** *=* ***z*** *+* ***f***

where:

- ***x*** = output vector of size *n,* where *n* is the number of industries
- ***z*** = intermediate demand and ***f*** = final demand with size *n*

For each industry, the value of the intermediate input required by all other industries to produce one unit of output is defined in the matrix of technological coefficients*,* ***A****(n,n)*. Expressing intermediate demand as the multiplication of the matrix of technological coefficients and the output vector in *Equation 1* yields:

*Equation 2*

***x*** *=* ***Ax*** *+* ***f***

Technology (*A*) is assumed to be fixed in the input–output framework, therefore, for any level of final demand (*f*), one can express the corresponding output vector (*x*) as:

*Equation 3*

***x*** *= (****I****–****A****) ^(–1)* ***f***

where:

- I is an identity vector of size (n,n)

If the Leontief inverse, *(****A****–****I****)^–1*, is non–singular, *Equation 3* determines the output level that exactly satisfies the final demand plus the corresponding intermediate demand that is needed for production. Consequently, calculating the impact of a change in final demand on output can be performed by applying *Equation 3* to differences:

*Equation 4*

***∆x*** *= (****I****–****A****) ^(–1)* ***∆f***

where:

- ***∆x*** = change in output level
- ***∆f*** = change in final demand

The calculated change in output will not only include the lost tourism income, but also the *indirect effect* of this decrease in exports: the decrease in output of all firms along the supply chain.

Assuming a constant ratio of value added to total output for each industry, the equation determines the change in GDP corresponding to the change in output including indirect effects. Estimates for the change in imports and employee compensation based on the assumption of a constant ratio of imports and compensation of employees to total output for each industry are also provided.

The technological coefficient matrix can be augmented with the household sector, which provides labor to other industries as their production input and receives wages as a payment for their service. Using this approach, the model estimates the *induced effects* on output, GDP, import, and the compensation of employees.

### Mapping of spending categories

This study assumed accommodations to correspond to the ‘hotels and lodging places,’ industry, food and beverages to ‘restaurants and drinking places,’ and medical care to ‘hospitals.’ Sightseeing was categorized to ‘other services,’ and included firms providing travel information, or arranging tours, or transportation for passengers. Local transport was distributed across ‘railways,’ ‘road passenger transport,’ ‘water transport services,’ and ‘air transport,’ in proportions informed by the Tourism Satellite Accounts[1]. Entertainment was allocated to ‘movie theatres,’ ‘libraries and museums,’ and ‘amusement and recreation’; proportionally to their relative importance in special exports [2]. Shopping was distributed across ‘agriculture,’ ‘foods manufacturing,’ ‘metal, metal products, and machinery,’ ‘other manufacturing,’ ‘paper industries and printing,’ ‘textile industry,’ and ‘retail trade’ proportionally to their relative importance in special exports [2].

### Statistical Information on Tourist Expenditure and the Informal Economy

Data provided by the Ministry of Tourism and Sports (MOTS) includes tourist expenditure which is captured via self–reported surveys; as such, informal sector spending is not differentiated and cannot be determined [3]. Nonetheless, since Thailand has a large informal economy, including family run businesses and the lack of a rigid system around employment registration and documentation, particularly for seasonal employees during peak tourism months, the proportion of informal spending is anticipated to be high [1]. The current analysis assumed the informal economy is represented in the spending statistics.

Tourism expenditure is documented by travelers exiting Thailand and conducted by face–to–face interviews at major international airports and border checkpoints [1, 4]

### Effect of dengue-endemic status on GDP due to reduced tourist arrivals

Besides assessing the GDP impact of a dengue outbreak, this study also estimated the influence of endemic dengue on GDP as an exploratory analysis. The assumed 12.4% decrease in international tourist arrivals from all countries (dengue endemic and non-endemic) (**S1 Table**) was based on a cross-country study exploring the effects of infectious disease eradication on international tourism flows and its national economic impact through tourism-related industries; the input value specific to dengue was used in this analysis [5]. The same study predicted an 18.3% increase in inbound tourism for Thailand assuming travel-related disease (dengue, ebola, malaria, and yellow fever) risk was eradicated. These values are substantially higher than the 4% impact on tourism assumed in the analysis estimating the impact of a dengue outbreak, as they correspond to the eradication of the disease as opposed to a decrease in incidence from an outbreak level to the incidence of an average year.

Rosselló *et al.* (2017) [5] repeated their analysis for tourist arrivals from developing countries and estimated a 40.2% decrease associated with endemic dengue; the relationship between decreases in tourist arrivals from developed source countries and dengue being endemic in the destination country was not statistically significant [5]. Of the two estimates provided by Rosselló *et al.* (2017) [5], this study used the more robust estimate related to international tourist arrivals from all countries (dengue–endemic and non–endemic). This estimate is based on a larger sample size, and the estimated results are more detailed. Therefore, this analysis assumed an estimated 12.4% decrease in international tourist arrivals due to endemicity of dengue.

This postulation estimated reduced tourism revenues by 7.64 billion USD and GDP by 2.94 billion USD (0.54%) from direct effects (**Table S5**). Based on the presumed reduction in tourist arrivals (**S1 Table**), the total impact of dengue on the overall decrease in GDP was 7.42 billion USD (1.36%) (**Table S5**). Further outcomes are presented in **Table S5**. As the standard assumptions of input-output models are more applicable to temporary dengue outbreaks rather than to long-term changes in dengue endemicity, these results are likely upward biased and constitute an upper limit for the tourism-related macroeconomic impact of dengue.

References

1. Thailand Ministry of Tourism & Sports. Thailand tourism satellite accounts. Inbound tourism expenditure by products and classes of visitors 2019 [cited 2022 10/27/]. Available from: <https://www.mots.go.th/more_news_new.php?cid=615>.

2. Buddhari A, Rugpenthum P, Thailand Bo. A better understanding of Thailand’s informal sector. Focus and Quick. 2019;156:1-13.

3. Economic Tourism and Sports Division, Permanent Secretary, Ministry of Tourism and Sports. Thailand's Tourism Statistics System 2019 [cited 2022 10/26/]. Available from: <https://www.mots.go.th/download/article/article_20191009135549.pdf>.

4. Noypayak W. Thailand’s international tourism expenditure survey 2006 [cited 2023]. Available from: <https://unstats.un.org/unsd/tradeserv/Workshops/Madrid/IWTS_Item17(Thailand).pdf>.

5. Rosselló J, Santana-Gallego M, Awan W. Infectious disease risk and international tourism demand. Health Policy Plan. 2017;32(4):538-48. Epub 2017/01/21. doi: 10.1093/heapol/czw177. PubMed PMID: 28104695.
