# Supplementary material for: The macroeconomic impact of a dengue outbreak: Case studies from Thailand and Brazil
Source: PLoS Negl Trop Dis. 2024 Jun 3;18(6):e0012201. doi: 10.1371/journal.pntd.0012201 (PMC11175482; doi:10.1371/journal.pntd.0012201)
Supplement: S2 Appendix — Estimation of lost workdays in the informal sector. (DOCX) [file pntd.0012201.s002.docx]

## S2 Appendix

### Inoperability and impact on Brazil’s GDP

For each industry, this study determined direct inoperability arising from the decrease in workforce due to dengue via *Equation 1*:

$\boldsymbol{Inoperability=}\frac{\boldsymbol{Lost work days}}{\boldsymbol{Total work days}}\boldsymbol{*}\frac{\boldsymbol{Compensation of employees}}{\boldsymbol{Output}}$ **(1)**

The ratio of lost workdays to total workdays expresses the proportion of the workforce absenteeism affected by the disruption. Total workdays were calculated by multiplying the number of employees in an industry by the total number of workdays in Brazil in 2019 minus the number of annual leave days. The compensation of employees paid by the industry, as reported in the Brazil National Accounts, represents the value created by its employees, and its ratio to output is used to proxy the proportion of output dependent on the workforce [1].

This direct inoperability was interpreted as an equivalent demand perturbation **c***.

To estimate the cascading effects arising from this initial perturbation, the interdependency matrix **A*** *Equation 2* was constructed as:

$\boldsymbol{A}^{\boldsymbol{*}}={\boldsymbol{[diag}\left( \boldsymbol{x} \right)\boldsymbol{]}}^{\boldsymbol{-1}}\boldsymbol{Z}$ **(2)**

where:

- **x** = vector of industry outputs of size *n*, **diag(X)** is a size (*n*,*n*) diagonal matrix containing elements of **x** in its diagonal; *n* being the number of industries;
- **Z** = size (*n*,*n*) matrix of interindustry intermediate consumption: **Z**_i,j_ is the value of intermediate inputs produced by industry *i* and used by industry *j*.

Inoperability including direct and indirect impact was calculated as Equation 3:

${\boldsymbol{q}=\left[ \boldsymbol{I-}\boldsymbol{A}^{\boldsymbol{*}} \right]^{\boldsymbol{-1}}\boldsymbol{c}}^{\boldsymbol{*}}$ **(3)**

where:

- **q** = inoperability vector of size *n*;
- **I** = identity matrix of size (*n*,*n*);
- ***c** =** demand perturbation vector of size *n*.

The impact on GDP was calculated by multiplying the impact on output by the ratio of GDP to output for each industry.

As pointed out by Dietzenbacher and Miller (2015) [2], this method is mathematically equivalent to interpreting the direct decrease in output due to productivity disruption as a change in final demand and deriving the impact on GDP using multipliers calculated based on the Leontief–inverse [2].

The assumed productivity loss per dengue case for employees was 10.7 days for hospitalized cases and 7.1 days for ambulatory cases [3]. For pediatric cases, the number of lost school days per dengue case (6.8 for hospitalized cases, and 5.2 days for ambulatory cases) was multiplied by the assumed ratio of sick children requiring a caregiver to forgo work [3].

An expansion factor of 2.03 was applied for the hospitalized setting to account for dengue underreporting. This expansion factor was calculated from a study conducted by Coelho *et al*. (2016) [4], who investigated the discrepancies between hospitalizations captured from the Brazil national surveillance system (SINAN) and the national hospitalization system (SIH/SUS). Data from the SIH/SUS expanded the estimate of dengue hospitalizations by more than 49.2% (35,016) hospitalizations in the 10 cities of the study compared with the data available from SINAN alone. This data was converted to an expansion factor of 2.03 for hospitalized cases in the model (based on a calculation of 1/0.492 = 2.03). Due to absence of similar reliable estimates for the expansion factor in an ambulatory setting, the expansion factor was derived by adjusting the expansion factor for the hospitalized setting by a factor of 2.0. This was based on the ratio between hospitalized and ambulatory expansion factors in Martelli *et al*. (2015) [5].

### Estimation of lost workdays in the informal sector

Employment data from Instituto Brasileiro de Geografia e Estatística reports the number of “persons employed” in each region, and also the “number of persons employed in formal employment [6].” Therefore, this study assumed that the number of “persons employed” also included workers engaged in informal employment. The difference between the two datasets was calculated and attributed to informal workers, and the share of informal workers was derived. The number of lost workdays was calculated among employees and caregivers on the total employed population and within the derived share of informal workers. The percentage of lost workdays in the informal sector was then calculated by dividing the lost workdays among informal workers by the lost workdays on the total population.

References

1. Santos JR. Inoperability input-output modeling of disruptions to interdependent economic systems. Syst Engin. 2006;9:20-34. doi: 10.1002/sys.20040.

2. Dietzenbacher E, Miller RE. Reflections on the inoperatility input-output model. Econ Syst Res. 2015;27(4):478-86.

3. Suaya JA, Shepard DS, Siqueira JB, Martelli CT, Lum LCS, Tan LH, et al. Cost of dengue cases in eight countries in the Americas and Asia: a prospective study. Am J Trop Med Hyg. 2009;80(5):846-55. Epub 2009/05/02. PubMed PMID: 19407136.

4. Coelho GE, Leal PL, de Paula Cerroni M, Simplicio ACR, Siqueira JB. Sensitivity of the dengue surveillance system in Brazil for detecting hospitalized cases. PLoS Negl Trop Dis. 2016;10(5):e0004705. doi: 10.1371/journal.pntd.0004705. PubMed PMID: 27192405; PubMed Central PMCID: PMCPMC4871568.

5. Martelli CMT, Siqueira JB, Parente MPPD, de Sene Amancio Zara AL, Oliveira CS, Braga C, et al. Economic impact of dengue: multicenter study across four Brazilian regions. PLoS Negl Trop Dis. 2015;9(9):e0004042. Epub 2015/09/25. doi: 10.1371/journal.pntd.0004042. PubMed PMID: 26402905; PubMed Central PMCID: PMCPMC4581827.

6. Instituto Brasileiro de Geografia e Estatística. Automatic Recovery System - SIDRA. Central Register of Companies. 2019 [cited 2022 10/3/]. Available from: <https://sidra.ibge.gov.br/pesquisa/cempre/quadros/brasil/2019>.
