# Supplementary material for: The macroeconomic impact of a dengue outbreak: Case studies from Thailand and Brazil
Source: PLoS Negl Trop Dis. 2024 Jun 3;18(6):e0012201. doi: 10.1371/journal.pntd.0012201 (PMC11175482; doi:10.1371/journal.pntd.0012201)
Supplement: S3 Appendix — (DOCX) [file pntd.0012201.s003.docx]

## S3 Appendix

### Analysis of the CEMPRE datasets

For each state in Brazil, the sum of lost workdays of patients and caregivers was distributed across industries in proportion to the distribution of employees across industries based on the Central Register of Companies (CEMPRE) datasets. The CEMPRE datasets reported state–level data on the number of employed persons by industry based on a sample of firms covering 53 million employees. These datasets only reported employment data for each industry if at least 3 companies contributed to the data in a state. This resulted in 22 missing entries out of 567 input values (4% of total observations and 0.5% in value). To address this, this study imputed the data for 18/22 values by first calculating the total number of employees missing per region in 2019 (total number of employees minus the sum of recorded employees by industry), and then assigning a share of the missing employees to the absent values based on the proportion consumed by the specific industry in the prior year where data were available. These data were used to estimate the industry distribution of dengue cases within each state, after normalizing it to match the industry distribution of employees reported in National Accounts data at the country level. For 4/22 missing values, data relating to their share were not available and were kept as missing. The number of jobs per industry reported in the National Accounts for Brazil was used to calculate the total number of working hours when determining the industry level inoperability.
