# Supplementary figures and images for: The macroeconomic impact of a dengue outbreak: Case studies from Thailand and Brazil
Source: PLoS Negl Trop Dis. 2024 Jun 3;18(6):e0012201. doi: 10.1371/journal.pntd.0012201 (PMC11175482; doi:10.1371/journal.pntd.0012201)

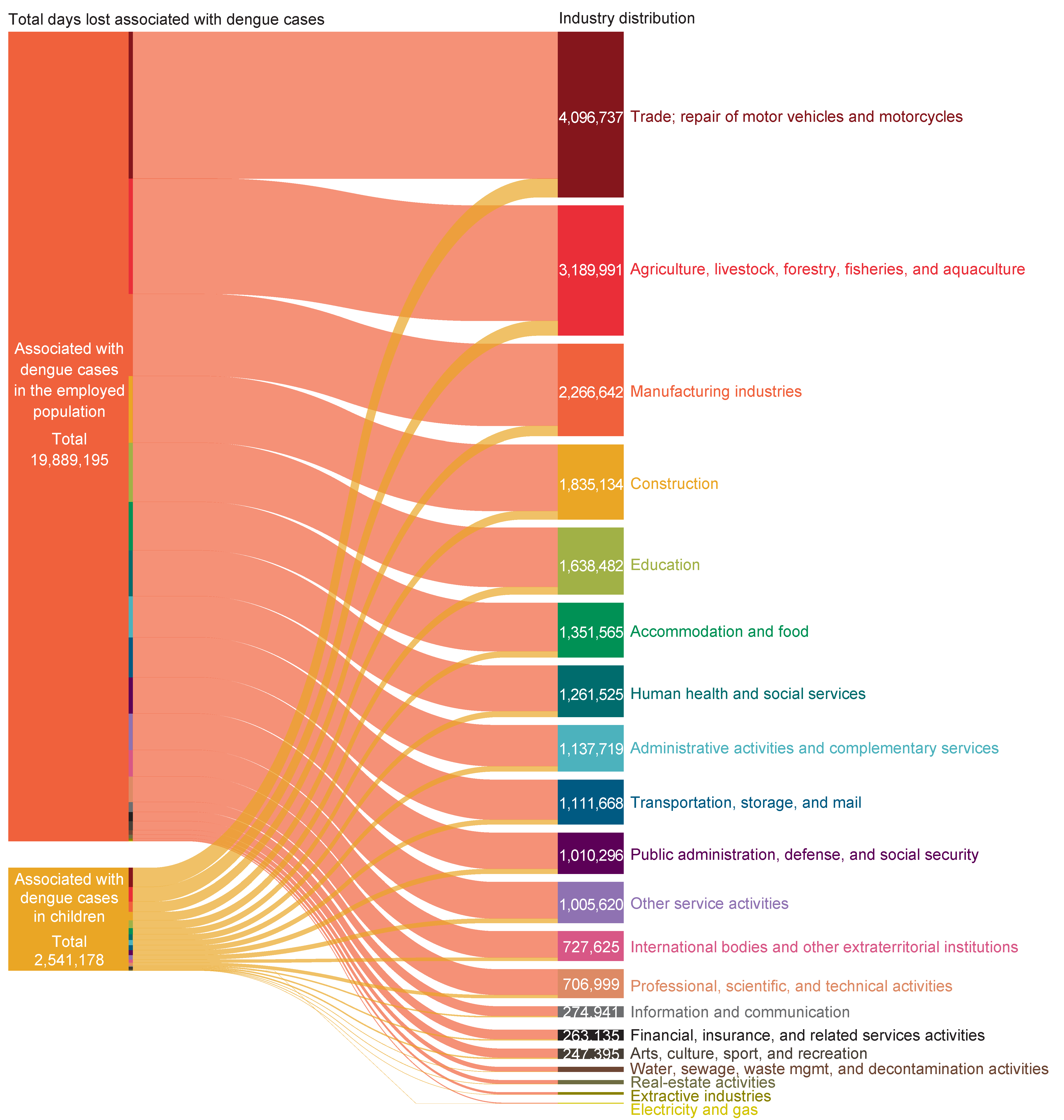

Supplement: S1 Fig — (TIF) [file pntd.0012201.s004.tif]

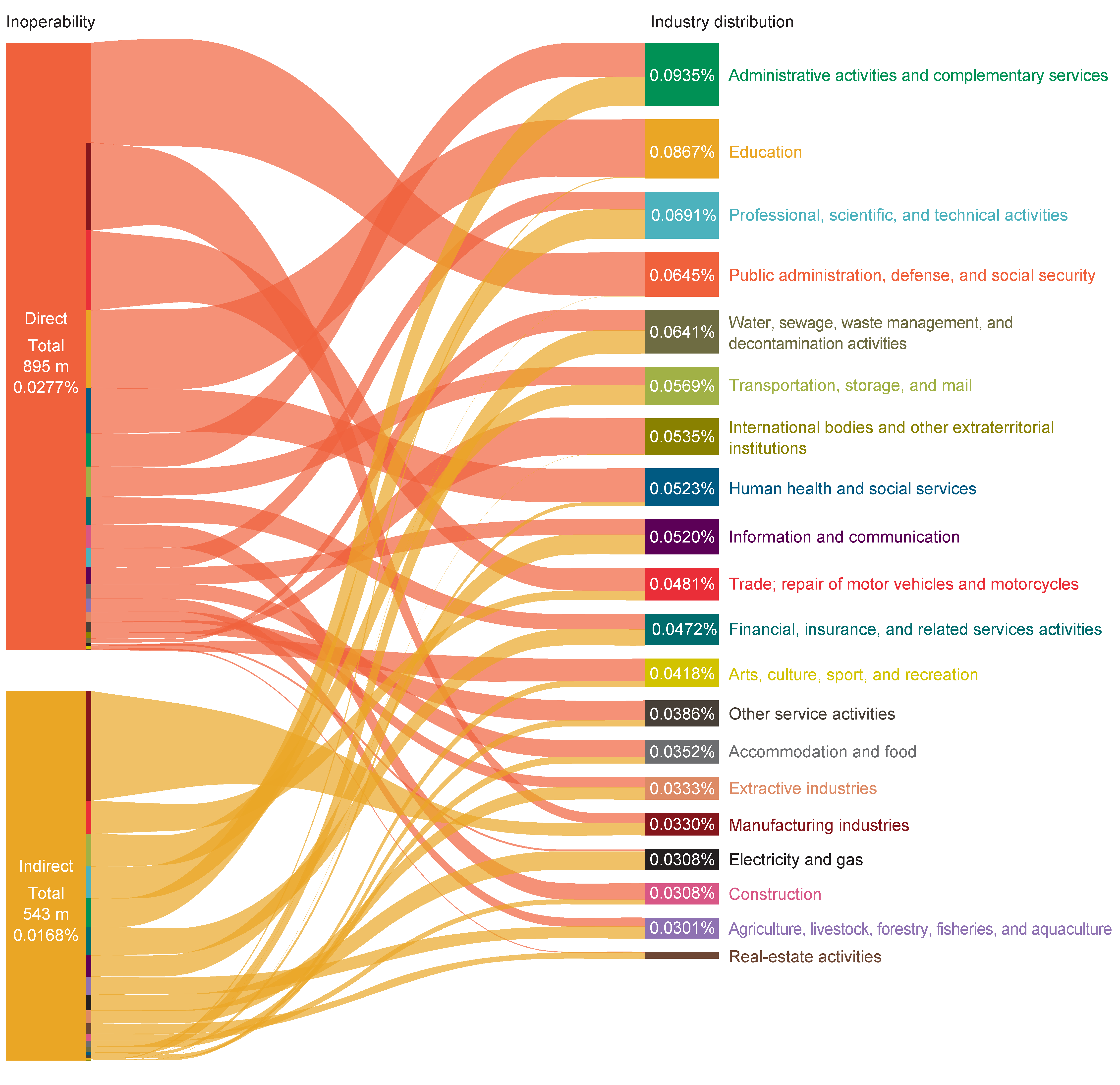

Supplement: S2 Fig — (TIF) [file pntd.0012201.s005.tif]

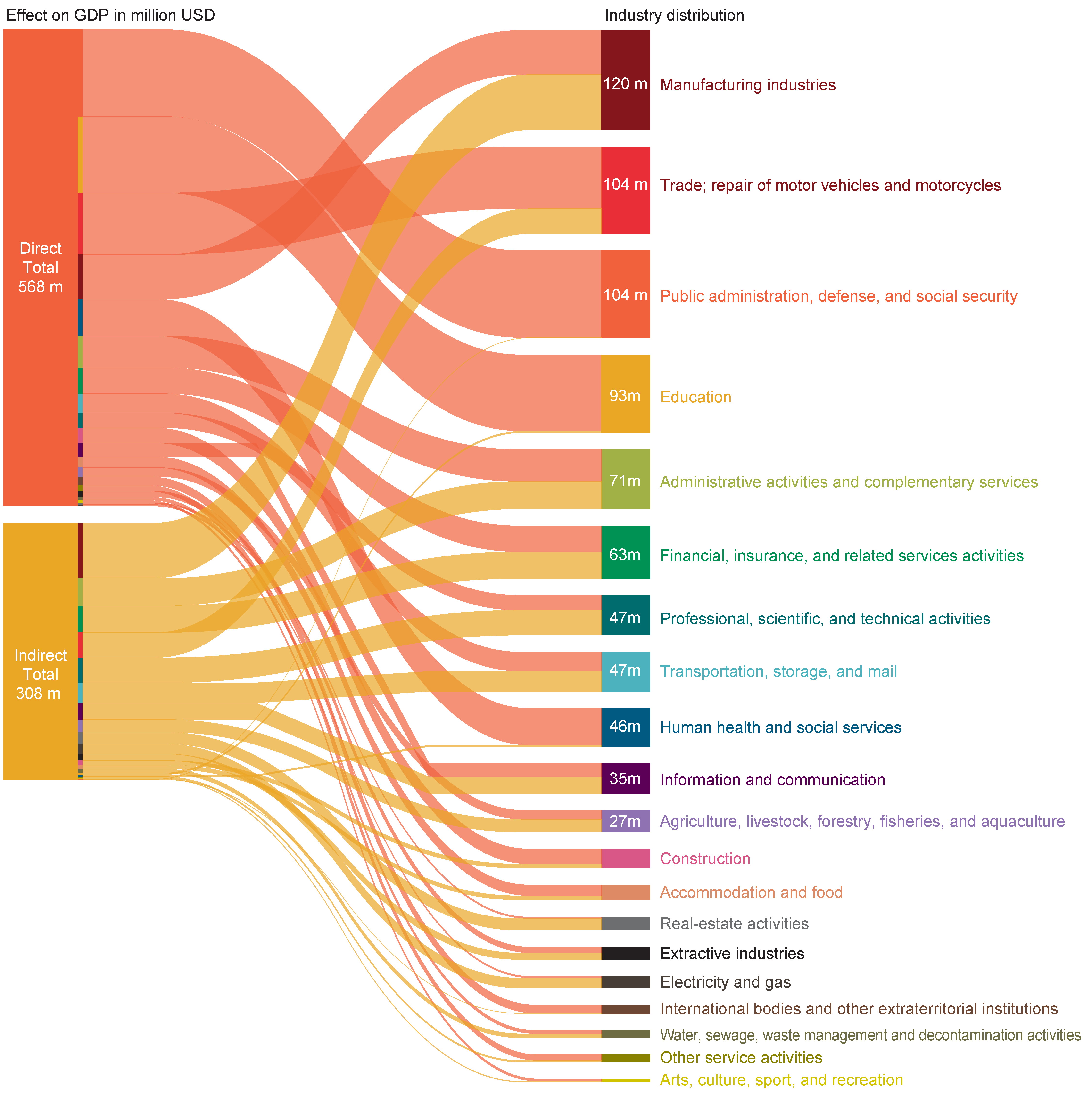

Supplement: S3 Fig — (TIF) [file pntd.0012201.s006.tif]

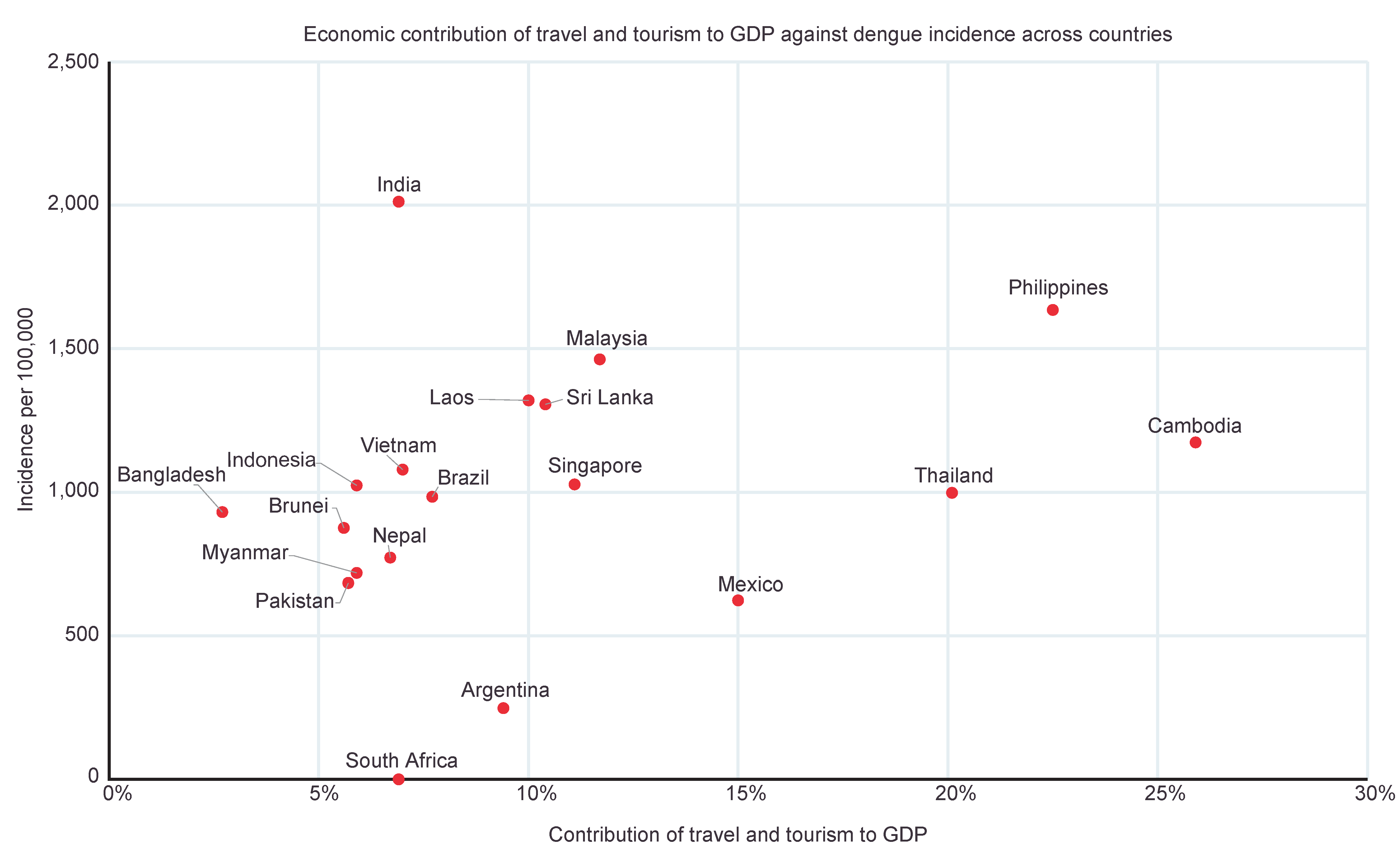

Supplement: S4 Fig — (TIF) [file pntd.0012201.s007.tif]
