# Supplementary material for: The macroeconomic impact of a dengue outbreak: Case studies from Thailand and Brazil
Source: PLoS Negl Trop Dis. 2024 Jun 3;18(6):e0012201. doi: 10.1371/journal.pntd.0012201 (PMC11175482; doi:10.1371/journal.pntd.0012201)
Supplement: S1 Table — (DOCX) [file pntd.0012201.s008.docx]

S1 Table. Input data for Thailand

| **Parameter** | **Value** | **Source** |
| --- | --- | --- |
| ***Decrease in the number of incoming international tourists*** | | |
| Non–endemic countries | 4% | Vasan *et al*. (2009) [1] |
| All countries  (endemic and non–endemic) | 12.4% | Rossello *et al*. (2017) [2] |
|  |  |  |
| ***Average share of spending categories in total international tourism spending in Thailand*** | | |
| Annual number of tourists arrivals^1^ | 39,916,251 | International tourist arrivals to Thailand (2019), Thailand Ministry of Tourism & Sports (arrivals and spending) [3] |
| Average spending per arrival by countries | See source | Thailand Ministry of Tourism & Sports report [4] |
|  |  |  |
| ***Assignment of international tourist spending by spending categories****^a,b^* | | |
| Shopping | 24% | Tourism receipts from international tourist arrivals by expenditure items (2019), Thailand Ministry of Tourism & Sports [4] |
| Entertainment | 9% |  |
| Sightseeing | 4% |  |
| Accommodation | 28% |  |
| Food and beverage | 21% |  |
| Local transport | 10% |  |
| Medical care | 2% |  |
| Miscellaneous | 2% |  |
|  | | |
| ***Assignment of international tourist spending by industries****^a.b^* | | |
| **Shopping** |  | Assumptions based on Tourism Satellite Accounts [4] and 2019 special  export as reported in the IO table of Thailand[5].  Shopping spending and Entertainment spending were split across the listed industries proportionally to special exports associated with these industries.  Local transport spending was split proportionally to 2019 tourism transport expenditures reported by means of transportation in the Tourism Satellite Accounts[4].  Miscellaneous spending was split across industries in the same proportion as total other spending |
| Agriculture | 5% |  |
| Foods manufacturing | 13% |  |
| Metal, metal products, and machinery | 8% |  |
| Other manufacturing | 27% |  |
| Paper industries and printing | 6% |  |
| Textile industry | 30% |  |
| Retail trade | 12% |  |
| **Entertainment** |  |  |
| Movie theatres | 2% |  |
| Amusement and recreation | 98% |  |
| **Sightseeing** |  |  |
| Other services | 100% |  |
| **Accommodation** |  |  |
| Hotels and lodging places | 100% |  |
| **Food and beverage** |  |  |
| Restaurants and drinking places | 100% |  |
| **Local transport** |  |  |
| Railways | 6% |  |
| Road passenger transport | 45% |  |
| Water transport services | 8% |  |
| Air transport | 41% |  |
| **Medical care** |  |  |
| Hospital | 100% |  |
|  |  |  |
| ***Input–output matrix*** | See source | Office of the National Economic and Social Development Council, Office of the Prime Minister [5] |
|  |  |  |
| ***Macroeconomic aggregates***  ***(millions of Baht)*** | See source | Office of the National Economic and Social Development Council, Office of the Prime Minister [6] |

^a^The model uses country level data for tourist spending structure by categories (see source).

^b^Accommodation (28%), shopping (24%), and food & beverage (21%) accounted for nearly 75% of the total average spending. Shopping constituted a larger share of spending for tourists arriving from Asian countries (e.g., 37% for visitors from Laos and 31% for visitors from China), but less so for European and American tourists (e.g., 12% for visitors from the United Kingdom, and 14% for visitors from the United States), who tended to spend more on local transport, accommodation, food & beverages, and sightseeing [7].

References

1. Vasan SS, Murtola TM, Mavalankar DV. Impact of the 2005‐2006 chikungunya outbreak on tourism revenues of French Réunion. WHO Dengue Bulletin (Special Supplement on Burden of Chikungunya and Dengue). 2009.

2. Rosselló J, Santana-Gallego M, Awan W. Infectious disease risk and international tourism demand. Health Policy Plan. 2017;32(4):538-48. Epub 2017/01/21. doi: 10.1093/heapol/czw177. PubMed PMID: 28104695.

3. Thailand Ministry of Tourism & Sports. International tourist arrivals to Thailand 2019 [cited 2022 9/9/]. Available from: <https://www.mots.go.th/mots_en> | article_20201103140333.xlsx (live.com).

4. Thailand Ministry of Tourism & Sports. Thailand tourism satellite accounts. Inbound tourism expenditure by products and classes of visitors 2019 [cited 2022 10/27/]. Available from: <https://www.mots.go.th/more_news_new.php?cid=615>.

5. Office of the National Economic and Social Development Council. Input-outputtables (I-O tables). 2015 [cited 2022 10/24/]. Available from: <https://www.nesdc.go.th/nesdb_en/ewt_news.php?nid=4429&filename=index>.

6. Office of the National Economic and Social Development Council. National income of Thailand. [cited 2022 10/26/]. Available from: <https://www.nesdc.go.th/nesdb_en/ewt_news.php?nid=4429&filename=index>.

7. Thailand Ministry of Tourism & Sports. Tourism receipts from international tourist arrivals by expenditure items 2019 [cited 2022 10/3/]. Available from: Ministry of Tourism and Sports (mots.go.th) | article_20201103140333.xlsx (live.com).
