# Supplementary material for: The macroeconomic impact of a dengue outbreak: Case studies from Thailand and Brazil
Source: PLoS Negl Trop Dis. 2024 Jun 3;18(6):e0012201. doi: 10.1371/journal.pntd.0012201 (PMC11175482; doi:10.1371/journal.pntd.0012201)
Supplement: S2 Table — (DOCX) [file pntd.0012201.s009.docx]

**S2 Table. Sensitivity analyses exploring alternative distributions of tourist spendings across industries in Thailand**

|  |  | **Estimated change in GDP** | | | |  |
| --- | --- | --- | --- | --- | --- | --- |
| **Alternative distribution of spending** |  | **Direct effect** | **Indirect effect** | **Induced effect** | **Direct + indirect effect** | **Total effect** |
| Base case | Million USD | –716 | –718 | –375 | –1,433 | –1,808 |
|  | Percentage | –0.13% | –0.13% | –0.07% | –0.26% | –0.33% |
| Share of Shopping is doubled, relative share of other spending categories unchanged | Million USD | –702 | –675 | –353 | –1,377 | –1,730 |
|  | Percentage | –0.13% | –0.12% | –0.06% | –0.25% | –0.32% |
| Share of Entertainment is doubled, relative share of other spending categories unchanged | Million USD | –775 | –684 | –375 | –1,459 | –1,834 |
|  | Percentage | –0.14% | –0.13% | –0.07% | –0.27% | –0.34% |
| Share of Sightseeing is doubled, relative share of other spending categories unchanged | Million USD | –722 | –718 | –378 | –1,440 | –1,818 |
|  | Percentage | –0.13% | –0.13% | –0.07% | –0.26% | –0.33% |
| Share of Accommodation is doubled, relative share of other spending categories unchanged | Million USD | –714 | –750 | –392 | –1,464 | –1,857 |
|  | Percentage | –0.13% | –0.14% | –0.07% | –0.27% | –0.34% |
| Share of Food and beverage is doubled, relative share of other spending categories unchanged | Million USD | –668 | –768 | –362 | –1,436 | –1,799 |
|  | Percentage | –0.12% | –0.14% | –0.07% | –0.26% | –0.33% |
| Share of Local transport is doubled, relative share of other spending categories unchanged | Million USD | –701 | –720 | –384 | –1,421 | –1,805 |
|  | Percentage | –0.13% | –0.13% | –0.07% | –0.26% | –0.33% |
| Share of Medical care is doubled, relative share of other spending categories unchanged | Million USD | –717 | –716 | –378 | –1,434 | –1,812 |
|  | Percentage | –0.13% | –0.13% | –0.07% | –0.26% | –0.33% |
| Share of Miscellaneous is doubled, relative share of other spending categories unchanged | Million USD | –715 | –718 | –375 | –1,433 | –1,808 |
|  | Percentage | –0.13% | –0.13% | –0.07% | –0.26% | –0.33% |
| **Alternative assignment of spending categories to industries** |  | **Direct effect** | **Indirect effect** | **Induced effect** | **Direct + indirect effect** | **Total effect** |
| 25% of “Food and beverage” spending assigned to Retail trade, another 25% to Foods manufacturing, remaining 50% to Restaurants and drinking places | Million USD | –771 | –680 | –374 | –1,451 | –1,825 |
|  | Percentage | –0.14% | –0.12% | –0.07% | –0.27% | –0.34% |
|  |  | **Direct effect** | **Indirect effect** | **Induced effect** | **Direct + indirect effect** | **Total effect** |
| 100% of “Shopping” spending assigned to Foods Manufacturing | Million USD | –694 | –788 | –380 | –1,482 | –1,862 |
|  | Percentage | –0.13% | –0.14% | –0.07% | –0.27% | –0.34% |
| 100% of “Shopping” spending assigned to Other Manufacturing | Million USD | –667 | –708 | –369 | –1,374 | –1,743 |
|  | Percentage | –0.12% | –0.13% | –0.07% | –0.25% | –0.32% |
| 100% of “Shopping” spending assigned to Textile industry | Million USD | –692 | –742 | –381 | –1,434 | –1,815 |
|  | Percentage | –0.13% | –0.14% | –0.07% | –0.26% | –0.33% |
| 50% of Entertainment assigned to Restaurants and drinking places, 50% to Amusement and recreation | Million USD | –681 | –742 | –373 | –1,422 | –1,795 |
|  | Percentage | –0.13% | –0.14% | –0.07% | –0.26% | –0.33% |

GDP, gross domestic product; USD, United States dollar.
