# Supplementary material for: The macroeconomic impact of a dengue outbreak: Case studies from Thailand and Brazil
Source: PLoS Negl Trop Dis. 2024 Jun 3;18(6):e0012201. doi: 10.1371/journal.pntd.0012201 (PMC11175482; doi:10.1371/journal.pntd.0012201)
Supplement: S3 Table — (DOCX) [file pntd.0012201.s010.docx]

S3 Table. Estimated effect of reduced tourist arrivals due to endemic dengue on macroeconomic outcomes in Thailand, 2019

|  | Effect (million USD) | | | | | Effect (%) | | | | |
| --- | --- | --- | --- | --- | --- | --- | --- | --- | --- | --- |
|  | **Direct effect** | **Indirect effect** | **Induced effect** | **Direct + indirect effect** | **Total effect** | **Direct** | **Indirect** | **Induced** | **Direct + indirect** | **Total** |
| GDP | –2,940 | –2,939 | –1,540 | –5,879 | –7,419 | –0.54 | –0.54 | –0.28 | –1.08 | –1.36 |
| Compensation of employees | –996 | –864 | –464 | –1,861 | –2,325 | –0.59 | –0.51 | –0.27 | –1.10 | –1.38 |
| Trade balance | –6,878 | 999 | 413 | –5,879 | –5,466 | –8.00 | 1.16 | 0.48 | –6.84 | –6.36 |
| Import | –756 | –999 | –413 | –1,756 | –2,169 | –0.37 | –0.48 | –0.20 | –0.85 | –1.05 |
| Export | –7,635 | 0 | 0 | –7,635 | –7,635 | –2.60 | 0.00 | 0.00 | –2.60 | –2.60 |

GDP, gross domestic product; USD, million United States dollar.
