# Supplementary material for: The macroeconomic impact of a dengue outbreak: Case studies from Thailand and Brazil
Source: PLoS Negl Trop Dis. 2024 Jun 3;18(6):e0012201. doi: 10.1371/journal.pntd.0012201 (PMC11175482; doi:10.1371/journal.pntd.0012201)
Supplement: S4 Table — (DOCX) [file pntd.0012201.s011.docx]

S4 Table. Input data for Brazil

| ***Regional employment statistics*** | | **Population** | **Dengue cases** | **Source** |
| --- | --- | --- | --- | --- |
| **Overall** | |  | 1,552,839 | IBGE, population estimates, 2019; IBGE, National Household Sample Survey, 2019 [1, 2] |
| Number of employees | | 94,956,159 |  |  |
| Working age population (aged 15–64 years) | | 168,365,259 |  |  |
| Percentage employed in working age population | | 56.4% |  |  |
| **North** | |  | 35,800 |  |
| Number of employees | | 7,474,593 |  |  |
| Working age population (aged 15–64 years) | | 13,732,287 |  |  |
| Percentage employed in working age population | | 54.4% |  |  |
| **Northeast** | |  | 213,700 |  |
| Number of employees | | 21,591,321 |  |  |
| Working age population (aged 15–64 years) | | 44,951,160 |  |  |
| Percentage employed in working age population | | 48.0% |  |  |
| **Central–East** | |  | 235,926 |  |
| Number of employees | | 7,891,909 |  |  |
| Working age population (aged 15–64 years) | | 12,877,549 |  |  |
| Percentage employed in working age population | | 61.3% |  |  |
| **Southeast** | |  | 1,018,611 |  |
| Number of employees | | 42,825,779 |  |  |
| Working age population (aged 15–64 years) | | 72,395,036 |  |  |
| Percentage employed in working age population | | 59.2% |  |  |
| **South** | |  | 48,802 |  |
| Number of employees | | 15,172,557 |  |  |
| Working age population (aged 15–64 years) | | 24,409,226 |  |  |
| Percentage employed in working age population | | 62.2% |  |  |
|  | |  |  |  |
| ***Distribution of employment across industries, by region*** | | See source | See source | IBGE SIDRA, Central Register of Companies, 2019 [3] |
| ***Distribution of dengue cases across age groups*** | |  |  |  |
| Children (aged 0–14 years) | | 16.8% |  | Ministry of Health data, 2019 [4] |
| Working age population (aged 15–64 years) | | 76.6% |  |  |
| Elderly (aged ≥65 years) | | 6.6% |  |  |
|  | |  |  |  |
|  | |  |  |  |
| ***Estimated number of dengue cases in Brazil*** | | **Distribution of cases across settings** | **Dengue cases** | **Source** |
| **Children (aged 0–14 years)** | |  | 1,031,269 | Ministry of Health data, 2019 [4] |
| Ambulatory | | 94.9% | 1,004,115 |  |
| Hospitalized | | 5.15% | 27,154 |  |
| **Working age population (aged 15–64 years)** | |  | 4,755,243^a^ |  |
| Ambulatory | Employed | 97.06% | 2,738,784 |  |
|  | Inactive and unemployed |  | 1,946,228 |  |
| Hospitalized | Employed | 2.94% | 41,056 |  |
|  | Inactive and unemployed |  | 29,175 |  |
| **Elderly (aged ≥65 years)** | |  | 401,833 |  |
| Ambulatory | | 92.13% | 385,996 |  |
| Hospitalized | | 7.87% | 15,837 |  |
| **Total** | |  | 6,188,345 |  |
| Ambulatory | |  | 6,075,122 |  |
| Hospitalized | |  | 113,223 |  |

| **Expansion factor** |  |  | Coelho *et al*. (2016); hospitalization adjusted by relationship drawn from Martelli *et al*. (2015) [5, 6] |
| --- | --- | --- | --- |
| Ambulatory | 2.03 |  |  |
| Hospitalized | 4.06 |  |  |
|  |  |  |  |
| **Number of workdays lost** |  |  |  |
| **Employees** |  |  | Suaya *et al*. (2009) [7] |
| Ambulatory | 7.1 |  |  |
| Hospitalized | 10.7 |  |  |
| **Caregivers** |  |  | Caregiver need for children: assumed to equal percentage of working age women in employment in data from IBGE*number of school days lost from Suaya *et al*. (2009), National Household Survey, 2019 [2, 7] |
| Ambulatory | 5.2 |  |  |
| Hospitalized | 6.8 |  |  |
|  |  |  | |
| ***Macroeconomic aggregates*** | | | |
| Output, GDP, compensation of employees, export, import, number of employees by industry; 2015 and 2019 | See source | See source | IBGE National Accounts [8] |
| Input–output matrix | See source | See source | IBGE National Accounts [8] |

^a^Sum total of the employed, inactive, and unemployed populations.

GDP, gross domestic product; IBGE, Instituto Brasileiro de Geografia e Estatístic

References

1. Sistema IBGE de Recuperação Automática - SIDRA. Cadastro Central de Empresas [cited 2022 10/24/]. Available from: <https://sidra.ibge.gov.br/pesquisa/cempre/quadros/brasil/2020>.

2. Instituto Brasileiro de Geografia e Estatística. National household sample survey. 2019 [cited 2022 10/3/]. Available from: <https://www.ibge.gov.br/en/statistics/social/population/18704-summary-of-social-indicators.html?edicao=31239&t=resultados>.

3. Instituto Brasileiro de Geografia e Estatística. Automatic Recovery System - SIDRA. Central Register of Companies. 2019 [cited 2022 10/3/]. Available from: <https://sidra.ibge.gov.br/pesquisa/cempre/quadros/brasil/2019>.

4. TabNet. Win32 3.0: DENGUE - Notificações registradas no Sistema de Informação de Agravos de Notificação - Brasil [cited 2022 10/26/]. Available from: <http://tabnet.datasus.gov.br/cgi/tabcgi.exe?sinannet/cnv/denguebr.def>.

5. Coelho GE, Leal PL, de Paula Cerroni M, Simplicio ACR, Siqueira JB. Sensitivity of the dengue surveillance system in Brazil for detecting hospitalized cases. PLoS Negl Trop Dis. 2016;10(5):e0004705. doi: 10.1371/journal.pntd.0004705. PubMed PMID: 27192405; PubMed Central PMCID: PMCPMC4871568.

6. Martelli CMT, Siqueira JB, Parente MPPD, de Sene Amancio Zara AL, Oliveira CS, Braga C, et al. Economic impact of dengue: multicenter study across four Brazilian regions. PLoS Negl Trop Dis. 2015;9(9):e0004042. Epub 2015/09/25. doi: 10.1371/journal.pntd.0004042. PubMed PMID: 26402905; PubMed Central PMCID: PMCPMC4581827.

7. Suaya JA, Shepard DS, Siqueira JB, Martelli CT, Lum LCS, Tan LH, et al. Cost of dengue cases in eight countries in the Americas and Asia: a prospective study. Am J Trop Med Hyg. 2009;80(5):846-55. Epub 2009/05/02. PubMed PMID: 19407136.

8. Instituto Brasileiro de Geografia e Estatística. System of national accounts. 2019 [cited 2022 10/3/]. Available from: <https://www.ibge.gov.br/en/statistics/economic/national-accounts/17173-system-of-national-accounts-brazil.html?edicao=32114&t=resultados>.
