# Supplementary material for: The macroeconomic impact of a dengue outbreak: Case studies from Thailand and Brazil
Source: PLoS Negl Trop Dis. 2024 Jun 3;18(6):e0012201. doi: 10.1371/journal.pntd.0012201 (PMC11175482; doi:10.1371/journal.pntd.0012201)
Supplement: S5 Table — (DOCX) [file pntd.0012201.s012.docx]

**S5 Table. Estimated industry distribution of international tourism revenue of Thailand in 2019**

| Industry | Tourism revenue from non–endemic countries | Tourism revenue from all countries |
| --- | --- | --- |
| Services | 63% | 61% |
| Transportation and communication | 15% | 14% |
| Textile industry | 7% | 7% |
| Other manufacturing | 6% | 7% |
| Foods manufacturing | 3% | 3% |
| Trade | 3% | 3% |
| Metal, metal products, and machinery | 2% | 2% |
| Paper industries and printing | 1% | 1% |
| Agriculture | 1% | 1% |
